# Complete and cooperative in vitro assembly of computationally designed self-assembling protein nanomaterials

**DOI:** 10.1038/s41467-021-21251-y

**Published:** 2021-02-09

**Authors:** Adam J. Wargacki, Tobias P. Wörner, Michiel van de Waterbeemd, Daniel Ellis, Albert J. R. Heck, Neil P. King

**Affiliations:** 1 Department of Biochemistry, University of Washington, Seattle, WA USA; 2 Institute for Protein Design, University of Washington, Seattle, WA USA; 3 Biomolecular Mass Spectrometry and Proteomics, Bijvoet Center for Biomolecular Research and Utrecht Institute for Pharmaceutical Sciences, Utrecht University, Utrecht, The Netherlands; 4 Graduate Program in Molecular and Cellular Biology, University of Washington, Seattle, WA USA

**Keywords:** Computational biophysics, Supramolecular assembly

## Abstract

Recent advances in computational methods have enabled the predictive design of self-assembling protein nanomaterials with atomic-level accuracy. These design strategies focus exclusively on a single target structure, without consideration of the mechanism or dynamics of assembly. However, understanding the assembly process, and in particular its robustness to perturbation, will be critical for translating this class of materials into useful technologies. Here we investigate the assembly of two computationally designed, 120-subunit icosahedral complexes in detail using several complementary biochemical methods. We found that assembly of each material from its two constituent protein building blocks was highly cooperative and yielded exclusively complete, 120-subunit complexes except in one non-stoichiometric regime for one of the materials. Our results suggest that in vitro assembly provides a robust and controllable route for the manufacture of designed protein nanomaterials and confirm that cooperative assembly can be an intrinsic, rather than evolved, feature of hierarchically structured protein complexes.

## Abstract (web summary)

Recent advances in computational methods have enabled the predictive design of self-assembling protein nanomaterials with atomic-level accuracy. Here authors investigate the assembly of two computationally designed, 120-subunit icosahedral complexes and find that assembly of each material from its two constituent protein building blocks was highly cooperative.

## Introduction

Many proteins assemble into multi-subunit complexes in order to perform highly specialized functions. The sophistication of these molecular machines has inspired efforts to adapt them to new purposes by making small alterations through mutation. This approach can lead to useful technologies that are inaccessible to other classes of materials^1,2^, but is fundamentally confined to the structural and functional space near to existing proteins.

Recently, a number of different approaches have proven capable of generating multi-subunit protein assemblies with well-defined structures. Two strategies have repeatedly achieved predictive positioning of protein subunits in three-dimensional complexes with atomic-level accuracy: the genetic fusion method^3,4^ and computational docking followed by protein–protein interface design^5–10^. This ability has opened up the possibility of tailoring the structures of multi-subunit complexes to specific functions, a long-standing goal in biotechnology. In fact, computationally designed protein assemblies are already being used as scaffolds for structure determination^11^ and multivalent antigen presentation^12–15^, and as containers for packaging and delivering biologics^16,17^. Continued advances in methods for designing, screening, and manufacturing novel nanomaterials will improve their performance in these applications and unlock additional utilities.

In addition to three-dimensional structure, the dynamics of the assembly process is a defining characteristic of a given multisubunit complex^18^. A general principle emerging from the study of many naturally occurring protein complexes is that they assemble with a high degree of cooperativity, which can be defined operationally by the absence of accumulated assembly intermediates and off-target products. In that sense, cooperative assembly processes are typically high-fidelity: they efficiently generate the target structure and are robust to perturbation. The combination of two features in particular appears to largely account for the observed cooperativity: (i) a hierarchical assembly process in which protein subunits first form oligomeric building blocks which then further assemble^19–21^ and (ii) specific, yet weak, protein–protein interactions between the building blocks^22,23^. This conclusion is supported both by experiment^24–29^ and by multiple theoretical models of assembly^30–33^. For example, a simple equilibrium model^21^ comprising a cascade of low-order reactions predicts that weak association energies are sufficient to drive the assembly of modest concentrations of oligomeric building blocks to completion, with very low levels of intermediate structures present at equilibrium. Kinetic models and accompanying experiments suggest deviation from cooperativity occurs when: (i) the nucleation of new assemblies is rapid relative to their completion, which causes subunit depletion and accumulation of on-pathway intermediates^27,30,34–36^; and (ii) the individual interactions between building blocks are too strong (i.e., insufficiently reversible), which causes kinetic trapping that locks errors into incomplete or alternative architectures^28,37,38^. Elegant studies combining bioinformatics and experimental characterization of assembly and disassembly have demonstrated that the evolutionary histories of self-assembling proteins recapitulate their assembly pathways^20^, and that both have been driven by the combination of the two critical features required for cooperativity defined above.

In contrast, the processes by which designed protein nanomaterials assemble remain incompletely characterized. Realizing the full potential of these materials will require sophisticated methods to monitor and understand the assembly process and how it may be manipulated. Here we investigate the assembly of two computationally designed two-component protein nanomaterials in detail using a combination of biochemical and biophysical methods as well as theoretical modeling. Our results answer important questions including the yield of the complete target structure relative to potential intermediates or off-target architectures and the robustness of the assembly process to perturbations in parameters such as subunit concentrations and stoichiometries. Furthermore, our detailed characterization of computationally designed protein complexes lacking an evolutionary history reveals aspects of protein assembly pathways that are intrinsic to stable multi-subunit complexes^39^.

## Results

### Variants of I53-40 and I53-50 assemble efficiently in vitro

**Fig. 1:**
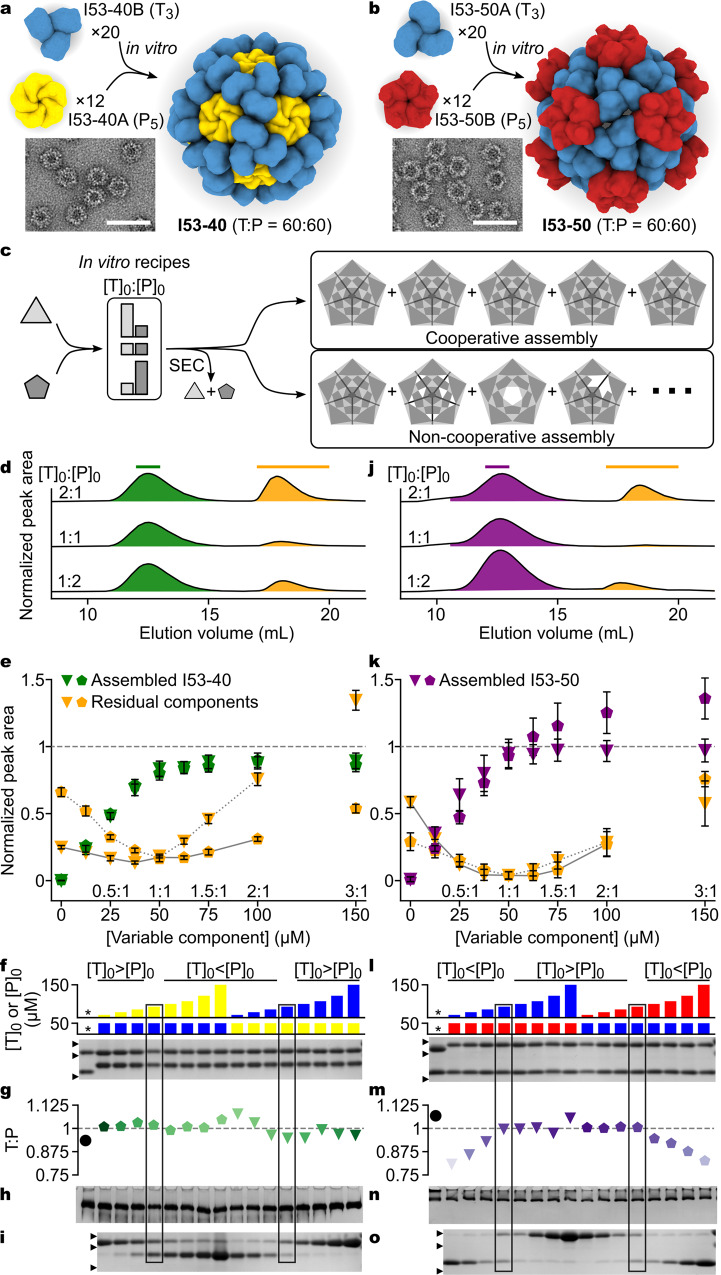
In vitro assembly analysis indicates cooperativity in most regimes. **a**–**b** Schematics of in vitro assembly and representative electron micrographs of in vitro-assembled nanomaterials. Scale bars, 50 nm. **c** Possible dependence of assembly structures (as Schlegel diagrams; transparent panes, voids) on initial subunit-stoichiometry. **d**, **j** Representative SEC traces from in vitro assembly reactions. Assembled nanomaterials and residual unassembled components were quantified by peak area (green, I53-40; purple, I53-50; orange, unassembled components). Green, purple, and orange bars indicate the fractions pooled for subsequent analyses. **e**, **k** Cooperativity analyses, in which peak area (normalized to the theoretical yield, gray dashed lines) is plotted against initial subunit stoichiometry. Data from reaction sets with variable [T]_0_ or [P]_0_ are shown using triangular or pentagonal symbols, respectively; the variable:constant component molar ratio is shown above the *x-*axis. Error bars represent the standard deviation of three technical replicates, and peak area is normalized to the average total signal obtained from six 50 μM equimolar assemblies. A characteristic chevron-like feature emerges when grouping assembly reactions initiated with sub-stoichiometric (dotted gray lines) or super-stoichiometric (solid gray lines) concentrations of pentamer. **f**, **l** SDS-PAGE analysis of nanomaterials assembled in vitro or in vivo (*) by co-expression in *E. coli*. Initial subunit ratios are indicated by colored bars ([T], blue; [I53-40P], yellow; [I53-50P], red); 25 kDa, 20 kDa, and 15 kDa marker locations are indicated by black arrows. **g**, **m** Analysis of the post-assembly T:P ratio in SEC-purified I53-40 (green) and I53-50 (purple) nanomaterials by PAGE gel densitometry. Data are normalized to the average ratio of signals (dashed line) in samples confirmed to be complete by native MS. Markers are shaded according to increasing initial [T]_0_:[P]_0_ ratio. **h**, **n** Non-denaturing PAGE of SEC-purified nanomaterials. **i**, **o** SDS-PAGE analysis of SEC-purified residual unassembled component fractions; 25 kDa, 20 kDa, and 15 kDa marker locations are indicated by black arrows. The datasets corresponding to equimolar ([T]_0_:[P]_0_ = 1) assembly reactions of I53-40 (**f**–**i**) and I53-50 (**l**–**o**) are enclosed in black boxes. Source data are provided as a Source Data file.

We focused our investigations on two recently reported two-component systems, I53-40 and I53-50 (ref. ^6^). Both nanomaterials are constructed from 20 trimeric (T_3_) and 12 pentameric (P_5_) building blocks. These are arranged along the three-fold and five-fold rotational axes in icosahedral point group symmetry, for a total of 60 copies of each monomeric subunit (Fig. 1a, b). In each nanomaterial, a single heteromeric, computationally designed protein-protein interface drives assembly of the pre-formed oligomeric building blocks to the icosahedral state. Although they share a common architecture, the two nanomaterials have unique atomic structures and interactions, and are derived from distinct but homologous naturally occurring building blocks. The trimeric components of I53-40 and I53-50 are bacterial aldolases (PDB IDs 4e38 and 1wa3, respectively), while the pentamers are archaeal and fungal riboflavin and lumazine synthases (PDB IDs 2b98 and 2obx, respectively). We engineered variants of the I53-40 components that were soluble when expressed and purified individually to enable controlled assembly of the nanomaterial in vitro (see Methods and Supplementary Table 1). When analyzed by size exclusion chromatography (SEC), each component eluted at the volume expected for its oligomeric state, while 50 μM mixtures of the components at a 1:1 molar ratio (all molar ratios calculated according to monomeric subunits, referred to as T and P for trimer-forming and pentamer-forming, respectively) yielded a predominant early peak corresponding to the expected icosahedral nanomaterial along with a small peak comprising residual, unassembled components (Supplementary Fig. 1a, b). For I53-50, we confirmed efficient in vitro assembly of an equimolar mixture of previously reported variants^6^ of the trimeric and pentameric subunits (Supplementary Fig. 1c, d and Supplementary Table 1). Dynamic light scattering (DLS) and negative stain electron microscopy (EM) analyses of both SEC-purified nanomaterials were consistent with assembly specifically to the designed icosahedral structures (Fig. 1a, b and Supplementary Fig. 1e, f).

### In vitro assembly at various subunit stoichiometries suggests cooperativity in most regimes

The two-component nature of I53-40 and I53-50 enabled us to investigate the cooperativity of in vitro assembly by analyzing reactions prepared at various subunit stoichiometries, an experiment we refer to as a “cooperativity analysis”. In a non-cooperative system, in vitro assembly reactions with stoichiometries far from equimolar should be dominated by partially assembled products (Fig. 1c). If instead assembly is highly cooperative, such reactions should produce fully assembled materials, without accumulation of partial assemblies and with excess component(s) remaining unassembled. We first used SEC to analyze two series of reactions for each nanomaterial in which the starting concentration of one component ([T]_0_ or [P]_0_) was held constant at 50 μM and the other component varied from 12.5 μM to 150 μM (0.25:1 to 3:1). Elution peaks corresponding to the assembled nanomaterials and residual unassembled components were baseline-separated and appeared at consistent elution volumes in all chromatograms (Fig. 1d, j and Supplementary Fig. 1a–d), hinting at the absence of small partial assemblies.

For I53-40, similar yields of assembled nanomaterial, measured by integrating peak areas in preparative SEC chromatograms, were observed at each subunit stoichiometry regardless of which component was held constant at 50 μM (Fig. 1e). The yield of assembled nanomaterial increased with limiting component until [T]_0_ = [P]_0_, followed by a small further increase approaching but not exceeding the expected theoretical yield at normalized peak area = 1. Correspondingly, the amount of residual, unassembled components in each reaction formed symmetrical chevron-like plots with minima near [T]_0_ = [P]_0_. The symmetry of this chevron-like feature is characteristic of efficient and cooperative assemblies, where incorporation of limiting components is virtually quantitative and all of the super-stoichiometric component remains unassembled.

SDS-PAGE analysis of the SEC-purified assembled nanomaterials from all I53-40 in vitro assembly reactions indicated that both components were present with no detectable variations in the relative intensities of the two bands (Fig. 1f–g). Native (non-denaturing) PAGE also revealed no differences, with each fraction yielding a single band that did not systematically vary in terms of migration distance (Fig. 1h). Likewise, DLS of the various SEC-purified nanomaterial fractions indicated hydrodynamic diameter was invariant at 22.7 ± 0.7 nm (Supplementary Fig. 1e). SDS-PAGE of the unassembled component fractions showed that they contained predominantly the excess component when subunit stoichiometries were far from equimolar, with a gradient toward equal amounts of both components when [T]_0_ = [P]_0_ (Fig. 1i). Together, these results suggested that the assembled nanomaterial was identical in composition across all input subunit stoichiometries, an outcome that is only expected if assembly is highly cooperative and the assembled material uniformly consists of complete, 120-subunit complexes.

Analysis of an initial set of I53-50 in vitro assembly reactions at various subunit stoichiometries revealed differences in assembly in two distinct regimes. In reactions where [T]_0_ ≥ [P]_0_, the yield of assembled nanomaterial matched the expectation of a cooperative assembly process and mirrored the data obtained for I53-40 (Fig. 1k). In contrast, reactions containing excess pentamer deviated from the behavior expected of a cooperative assembly process. Specifically, higher protein absorbance was observed in the assembled nanomaterial peaks, and less in the residual unassembled component peaks, than expected. This effect was clearest in reactions where [P]_0_ >50 μM, where the peak area corresponding to the assembled nanomaterial continued to increase with increasing [P]_0_ instead of plateauing, but also manifested at [P]_0_ <50 μM as an asymmetric distortion in the shape of the chevron-like residual component plots, presumably due to over-incorporation of P into high MW species (Fig. 1k and Supplementary Fig. 1c, d).

SDS-PAGE analysis of the products of the various I53-50 in vitro assembly reactions corroborated the two different assembly regimes observed by SEC. Specifically, the reactions where [T]_0_ ≥ [P]_0_ yielded nanomaterials comprising both components with no detectable variations in the relative intensities of the two bands (Fig. 1l, m), consistent with the idea of a cooperative assembly process yielding complete, 120-subunit nanomaterials. However, nanomaterials isolated from assembly reactions containing excess pentamer showed subtle yet systematic deviations in relative band intensities, with decreased T:P relative band intensity correlating with increased [P]_0_ in the assembly reaction. Differences in the assembled nanomaterials were not apparent by native PAGE, which yielded a single band with a slight trailing smear in all samples (Fig. 1n). DLS was also unable to distinguish between SEC-purified I53-50 from the various in vitro assembly reactions, yielding an average diameter of 22.6 ± 1.4 nm (Supplementary Fig. 1f). SDS-PAGE of the unassembled component fractions revealed a pattern very similar to that of I53-40 except that less of the limiting component remained unassembled for I53-50, indicating that I53-50 assembles more efficiently than I53-40 at the tested concentrations when the input subunit stoichiometry is near equimolar (Fig. 1o). Overall, the data for I53-50 suggested that assembly is cooperative where [T]_0_ ≥ [P]_0_, while aberrant assemblies are formed when P is super-stoichiometric. The uniformity of the SEC, native PAGE, and DLS data is inconsistent with the formation of off-pathway architectures, suggesting that the aberrant assemblies are either incomplete (most likely lacking trimeric building blocks) or complete, 120-subunit complexes that encapsulate or bind extra pentameric components.

### Determination of the precise composition of I53-40 and I53-50 assemblies by native mass spectrometry

**Fig. 2:**
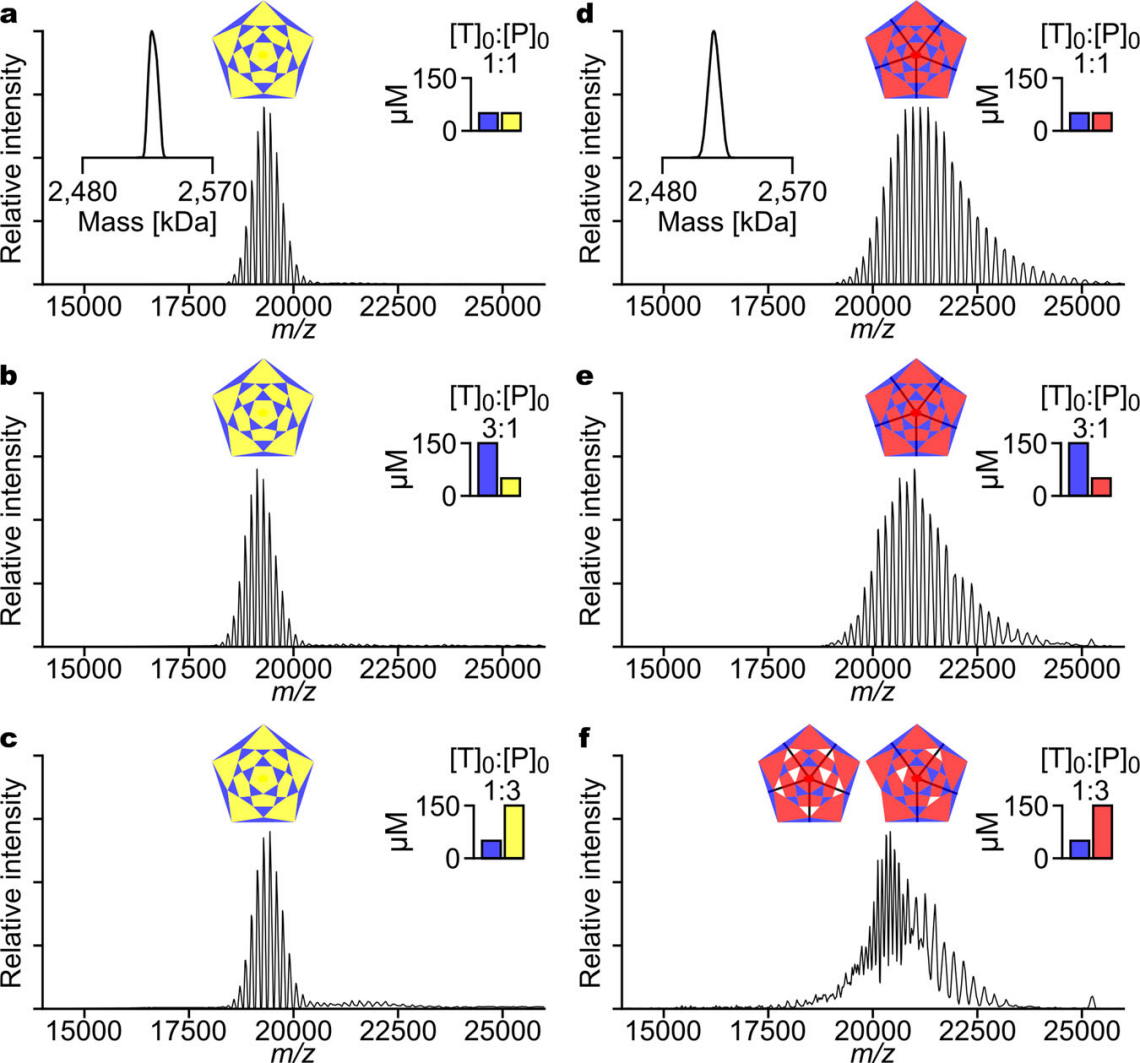
Determination of the precise composition of I53-40 and I53-50 assemblies by native mass spectrometry. Native mass spectra of SEC-purified I53-40 assemblies (**a**–**c**) and I53-50 assemblies (**d**–**f**). The subunit stoichiometry of the in vitro assembly reaction that produced each material is indicated by the bar plot insets. Insets in **a** and **d** show the deconvoluted masses of the materials purified from reactions where [T]_0_ = [P]_0_. Assemblies deriving from unpurified assembly reactions were analyzed in **a**–**d**, whereas **e**–**f** show assembly reaction products after SEC purification. The mass spectrum for the I53-50 [T]_0_:[P]_0_ = 1:3 assembly reaction (**f**) reveals peak series from at least two different species, neither of which overlaps with the complete architecture. Diagrams show representations of complete and hypothetical partial nanomaterials lacking trimeric building blocks. Source data are provided as a Source Data file.

To complement the results observed in our biochemical analyses and gain greater insight into the precise nature of the assembly products, we investigated the composition of the assembled materials at high resolution using native mass spectrometry, a method quite suitable to study the assembly of viruses and other large complexes^40,41^. I53-40 was found to assemble cooperatively, forming the complete 120-subunit architecture at all component ratios tested (Fig. 2a–c). I53-50 was also confirmed to assemble cooperatively under equimolar conditions (Fig. 2d) and for SEC-purified samples at 3:1 [T]_0_:[P]_0_ (Fig. 2e). However, when [T]_0_:[P]_0_ was 1:3, a mixture of species that did not include the complete architecture was observed (Fig. 2f). These results were further validated by tandem MS experiments allowing in-depth characterization and unambiguous mass determination (see assignments of tandem MS experiments in Supplementary Fig. 2 and Supplementary Table 2).

### Biochemical and biophysical characterization of nanomaterial stability and composition

We further analyzed the assembled materials using several biochemical and biophysical techniques, with two goals. First, we wished to determine whether these widely accessible methods could corroborate the constant (I53-40) and detect the variable (I53-50) compositions of the nanomaterials prepared from in vitro assembly reactions at various subunit stoichiometries. Second, we aimed to establish a suite of assays for characterizing the stability of these nanomaterials, an important attribute for manufacturing and functionalization.

**Fig. 3:**
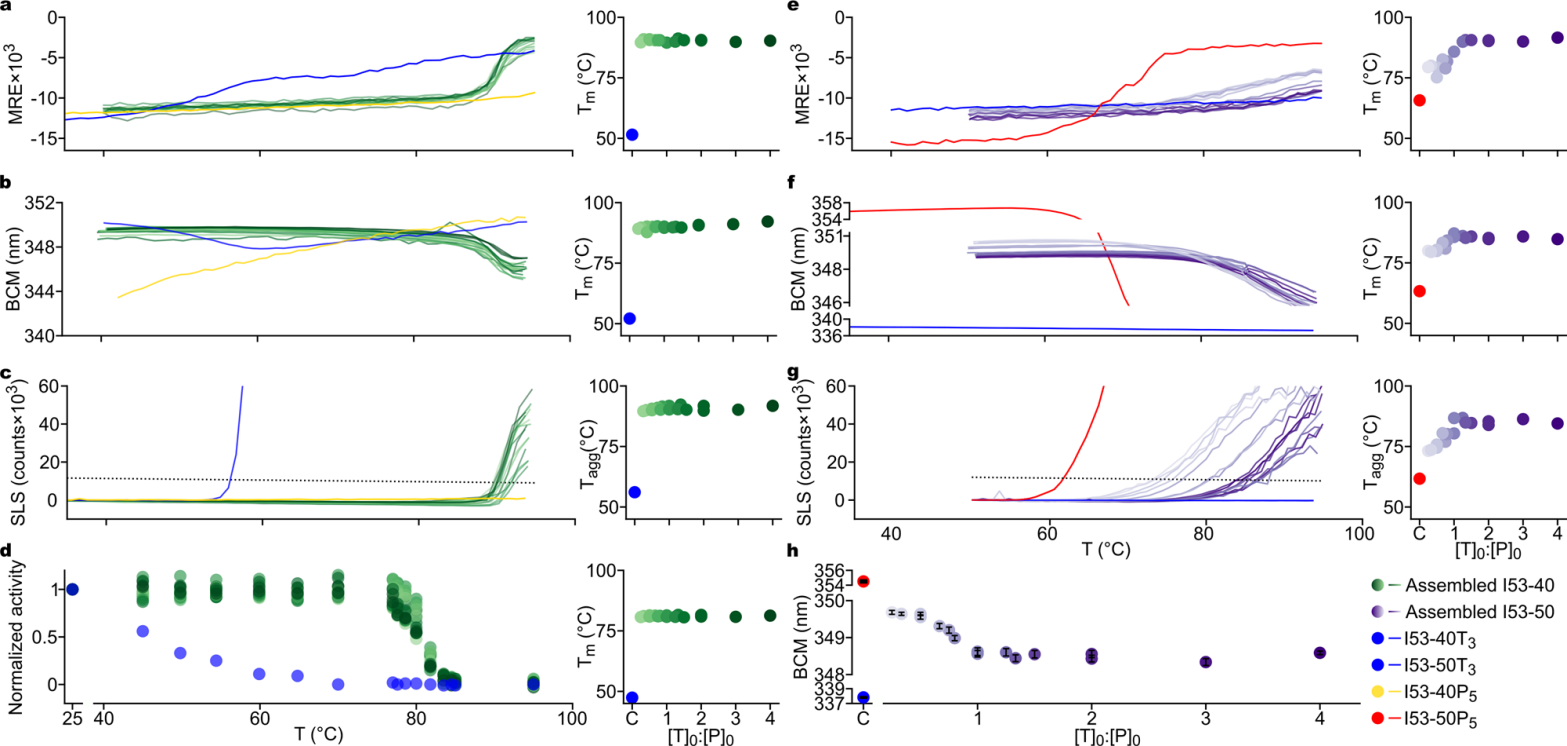
Biochemical and biophysical characterization of nanomaterial stability and composition. Representative samples of SEC-purified I53-40 and I53-50 were monitored by **a**, **e** CD reported as MRE (mean residue ellipticity; deg × cm^2^/dmol) at 222 nm; **b**, **f** intrinsic fluorescence, plotted as the barycentric mean (BCM) of the emission spectrum; and **c**, **g** SLS as a function of temperature. **d** Aldolase activity in I53-40 and I53-40T_3_ samples after incubation at selected temperatures for 1 h, normalized to activity in samples incubated at 25 °C. **h** Ambient temperature fluorescence emission (average BCM) for SEC-purified I53-50 samples plotted as a function of [T]_0_:[P]_0_ ratio. Error bars represent the standard deviation of five scans. For all panels, marker and line saturation correlate with increasing [T]_0_:[P]_0_ ratio at mixing, and adjacent dot plots track *T*_m_ or *T*_agg_ (determined by second derivative analysis or by the intersection of each trace with a defined threshold; dotted lines) of each SEC-purified nanomaterial as a function of [T]_0_:[P]_0_ ratio. Source data are provided as a Source Data file.

Thermal melts of the individual components of I53-40 and I53-50 monitored by circular dichroism (CD) showed that I53-40P_5_ and I53-50T_3_ did not unfold at temperatures up to 95 °C, consistent with their derivation from proteins from hyperthermophilic organisms^42,43^, while I53-40T_3_ and I53-50P_5_ exhibited melting temperatures (*T*_m_) of 55 and 70 °C, respectively (Fig. 3a, e and Supplementary Fig. 3a, b). Thermal melts of icosahedral I53-40 and I53-50 produced by co-expression in *Escherichia coli* or in vitro assembly at equimolar subunit stoichiometry revealed substantial stabilization of I53-40T_3_ and I53-50P_5_ in the context of each assembled nanomaterial. The *T*_m_ of I53-40 was approximately 90 °C, 35 °C above the *T*_m_ of I53-40T_3_, while I53-50 only began to lose ellipticity around 90 °C, representing an increase in *T*_m_ of >20 °C compared to I53-50P_5_. Given that increases in *T*_m_ of only a few degrees are considered a success in evolution-based protein engineering strategies^44–47^ and can have important technological implications, these data support the notion that incorporation of protein subunits into higher-order complexes is a general and effective stabilization strategy^48^.

We used CD, intrinsic tryptophan fluorescence, and static light scattering (SLS) to monitor the thermal denaturation of I53-40 complexes assembled in vitro at various subunit stoichiometries, and observed effectively no differences in melting temperature (*T*_m_; Fig. 3a, b) or in the onset of aggregation (*T*_agg_; Fig. 3c). Moreover, incubating I53-40 samples at various temperatures for 1 h before returning them to room temperature and assaying them for the aldolase activity of the trimeric subunit (see “Methods”) showed that the assembled nanomaterial samples uniformly lost 50% of their activity after incubation at 80 °C, while the free trimeric component lost 50% of its activity after incubation at 45 °C (Fig. 3d). These data again confirmed that the I53-40 assembly reaction produces a single, homogeneous product at any input subunit stoichiometry and that the trimeric component is substantially stabilized upon assembly.

In contrast, CD, intrinsic fluorescence, and SLS of I53-50 complexes assembled in vitro at various subunit stoichiometries suggested the existence of two distinct groups of assembled materials. Materials from assembly reactions where [T]_0_ < [P]_0_ exhibited a range of melting and aggregation temperatures correlating with increasing [T]_0_:[P]_0_, while *T*_m_ and *T*_agg_ were high and invariant where [T]_0_ ≥ [P]_0_ (Fig. 3e–g). Intrinsic fluorescence proved capable of distinguishing the various I53-50 complexes even without heating, as the trimeric and pentameric components of I53-50 have very different fluorescence emission properties on account of a solvent-exposed tryptophan in the I53-50 pentamer^12^. This feature led to systematic deviations in the fluorescence emission spectra of complexes from assembly reactions where [T]_0_ < [P]_0_, indicating these SEC-purified complexes contained more P subunits relative to T subunits than complexes prepared at [T]_0_ ≥ [P]_0_ (Fig. 3h). It is noteworthy that all four assays showed a clear demarcation between I53-50 assembled at [T]_0_:[P]_0_ ratios <1 and ≥1. Together, these results support and extend the SEC, PAGE, and native MS data presented in Figs. 1 and  2 showing that when [T]_0_ ≥ [P]_0_, assembly appears to be cooperative and a single assembly product dominates. They also indicate that the aberrant assemblies obtained when [T]_0_ < [P]_0_ can be distinguished by differences in physical stability and fluorescence emission.

Finally, we used SEC to examine the stability of in vitro-assembled I53-40 in response to dilution, as studies of several virus capsids have demonstrated kinetic trapping of complete assemblies that results in a marked hysteresis of disassembly^24,26,49^. In assembly reactions containing a low concentration of components ([T]_0_ = [P]_0_ = 5 μM), assembled I53-40 never exceeded a mass fraction of 20%, a value approached after incubation for ~100 h (Supplementary Fig. 4). In contrast, 100 μM assembly reactions proceeded to completion (>80% mass fraction assembled nanomaterial) overnight, and subsequent dilution to 5 μM did not trigger disassembly over 750 h. We were unable to obtain similar data for I53-50 because it assembled efficiently even at the low concentration detection limit of SEC. The resistance to disassembly upon dilution we observed for I53-40 could be an important property for applications such as multivalent antigen presentation or biologics delivery, where the materials will be substantially diluted upon administration in vivo.

### A simple equilibrium model enables estimation of thermodynamic parameters and differentiates cooperative and non-cooperative regimes

Although experimentally determining the association energies of individual interactions in self-assembling systems is difficult, comparing theoretical models of virus capsid assembly to experimental data measuring the efficiency of assembly has been useful for estimating interaction energies^23,50^. To estimate the affinity of the computationally designed protein–protein interfaces in I53-40 and I53-50 and to determine whether simple models could account for the cooperative and non-cooperative regimes we observe, we applied Twarock and coworkers’ extension^51^ to Zlotnick’s equilibrium model for virus capsid assembly^21^. In their generalized model, which enables the analysis of multi-component systems, the equilibrium concentration of any species in a hierarchically assembling system is related to: (i) the equilibrium concentration of free oligomeric component(s), (ii) the local bonding structure of the components in the context of the assembly, and (iii) the individual contact energies (Δ*G*_con_) between the components. Several features of our experimental system enabled simplifications to the generalized model. Specifically, (i) the assembled nanomaterial and unassembled building block concentrations are quantifiable, (ii) the stoichiometry and symmetry of the closed architecture is known, and (iii) assembly of the icosahedral I53-40 and I53-50 complexes is driven by a single heteromeric interaction, neither trimer–trimer nor pentamer–pentamer interactions contribute substantially. Applying these simplifications (see “Methods”), the equilibrium concentrations of fully assembled I53-40 or I53-50 and residual unassembled components can be related to starting component concentrations by the equations:

1 $$[{\mathrm{T}}_{60}{\mathrm{P}}_{60}] = \left( {\frac{{3^{20} \times 5^{12} \times \left( {\frac{{[{\mathrm{T}}]}}{3}} \right)^{20} \times \left( {\frac{{[{\mathrm{P}}]}}{5}} \right)^{12} \times e^{\left( {60\, \times\, {{\Delta }}G_{{\mathrm{con}}}/ - R \times 296\,{\mathrm{K}}} \right)}}}{{60}}} \right)$$

2 $$[{\mathrm{T}}]_0 = 60[{\mathrm{T}}_{60}{\mathrm{P}}_{60}] + [{\mathrm{T}}]$$

3 $$[{\mathrm{P}}]_0 = 60[{\mathrm{T}}_{60}{\mathrm{P}}_{60}] + [{\mathrm{P}}]$$

where T_60_P_60_ is the assembled icosahedral complex, T and P are single subunits of unassembled trimeric and pentameric building blocks, and [T]_0_ and [P]_0_ are starting concentrations of the building blocks. The only free parameter remaining in the equation (other than temperature (296 K), which, like [T_60_P_60_], [T], and [P], is experimentally measurable) is Δ*G*_con_, the average per-contact association energy.

Similar equations can be used to predict the concentrations of on-pathway intermediates as a function of [T]_0_ and [P]_0_ (see “Methods”). These systems of equations predict infinitesimally low concentrations of on-pathway intermediates when [T]_0_ and [P]_0_ are near equimolar (Supplementary Fig. 5a, b), and only low to moderate concentrations of intermediates over a broad range of divergent stoichiometries and Δ*G*_con_ values (Supplementary Fig. 5c, f). A single intermediate containing one trimeric void (T_57_P_60_) was the only non-target species predicted in any significant quantity for non-stoichiometric assemblies (as much as 10% by mass of assembled product), appearing across a range of Δ*G*_con_ only when [P]_0_ > [T]_0_ (Supplementary Fig. 5d, f). Therefore, a simplified approach considering only Eqs. (1–3) above was employed for analysis of equimolar data series (see below), where intermediates are not expected to accumulate. A more inclusive approach considering the equilibrium concentrations of several intermediate species was employed for the cooperativity analyses, where partial assemblies were experimentally observed for I53-50.

**Fig. 4:**
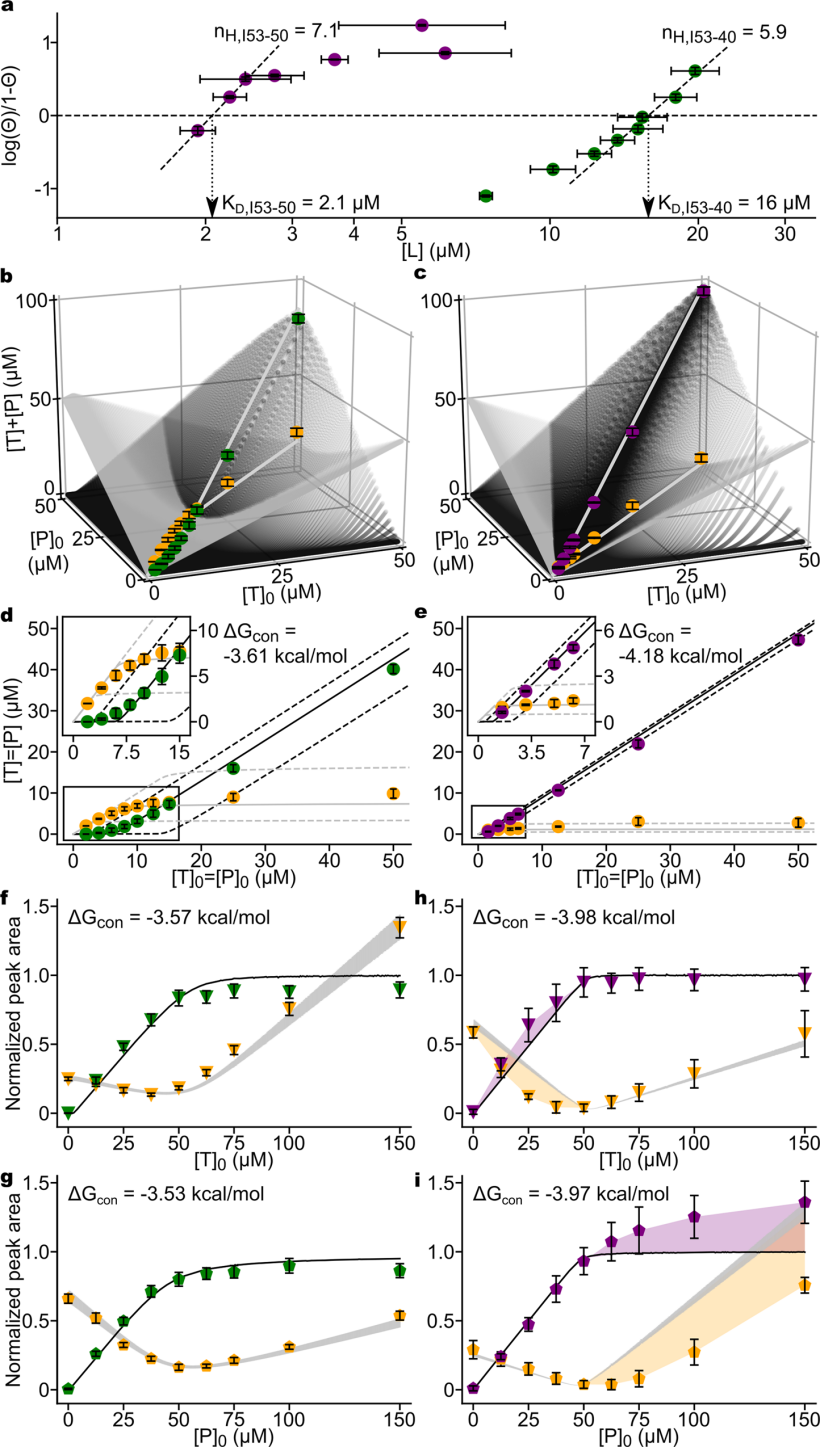
Comparison of experimental in vitro assembly data to a simple equilibrium model. For all panels, experimental data are plotted in green (assembled I53-40), purple (assembled I53-50), and orange (unassembled components). For all plots, error bars are standard deviations of three assembly replicates. **a** Quantitative analysis of equimolar assemblies are represented as Hill plots (Θ, assembly efficiency; L, free components). Hill coefficients are equal to the slopes of dashed regression lines, whose *x*-intercepts (dotted drop-lines with arrows) approximate the apparent K_D_s of assembly. Partitioning into high and low MW species is plotted in 3D space for **b** I53-40 and **c** I53-50 assemblies as a function of input [component]. This inclusive equilibrium model considers several nearly complete structures (black surface scatters, see “Methods”). Likewise, the smallest on-pathway species are summed with [T] and [P] (silver surface scatters). Gray lines emphasize output of the inclusive model at equimolar concentrations. **d**, **e** The same data as above, with the simplified equilibrium model in the 2D [T]_0_ = [P]_0_ plane. Only complete 120-subunit architectures (black) and free components (silver) are considered, and therefore plotted on axes where T = P. Solid lines represent the model output at the indicated best fit Δ*G*_con_ and dashed lines at best fit ±0.25 kcal/mol. Insets highlight the agreement between data and model at low [T]_0_ = [P]_0_. Cooperativity analyses for **f**, **g** I53-40 and **h**, **i** I53-50 report average peak area as a function of [variable component]. Raw signals are normalized to equimolar assemblies. The inclusive equilibrium models were modified to plot relative peak area of high MW (black lines) and low MW fractions (silver lines) as a function of [variable component]. The silver line-thickness is equal to twice the standard deviation associated with experimentally determined signal factors (see “Methods”). The values for best fit Δ*G*_con_ are indicated. For determination of Δ*G*_con_ for I53-50, only data where initial [T]_0_ ≥ [P]_0_ were considered. In **h**, **i**, the purple and orange colored areas emphasize the difference between inclusive equilibrium models and experimental data when [P]_0_ > [T]_0_. Source data are provided as a Source Data file.

We gathered additional data on the efficiency of in vitro assembly of I53-40 and I53-50 over a range of input subunit concentrations at [T]_0_ = [P]_0_, analogous to datasets previously fit to theoretical models of virus capsid assembly. The Hill coefficients calculated from these data were 5.9 for I53-40 and 7.1 for I53-50, indicating a high degree of cooperativity (Fig. 4a). Numerically sampling Δ*G*_con_ enabled us to fit the predicted values for species concentration to those observed experimentally in these equimolar in vitro assembly reactions, as well as the reactions in the cooperativity analyses presented in Fig. 1 (Fig. 4b–i and Supplementary Fig. 5a). Percent error minimization converged on Δ*G*_con_ values of about 3.6 kcal/mol for I53-40 and 4.1 kcal/mol for I53-50, which, intriguingly, are similar to values previously observed for cooperatively assembling virus capsids^23,33,50,52^. Overlaying the experimental data onto the model shows that while the I53-40 data globally matches the model well, the I53-50 data deviate from the model in the regime where pentamer is super-stoichiometric (Fig. 4h–i). However, reactions at [T]_0_ ≥ [P]_0_ matched the expected yields of high and low MW species, consistent with our observations of cooperative and complete assembly in this regime.

### Incomplete I53-50 assemblies can be cured by component addition

**Fig. 5:**
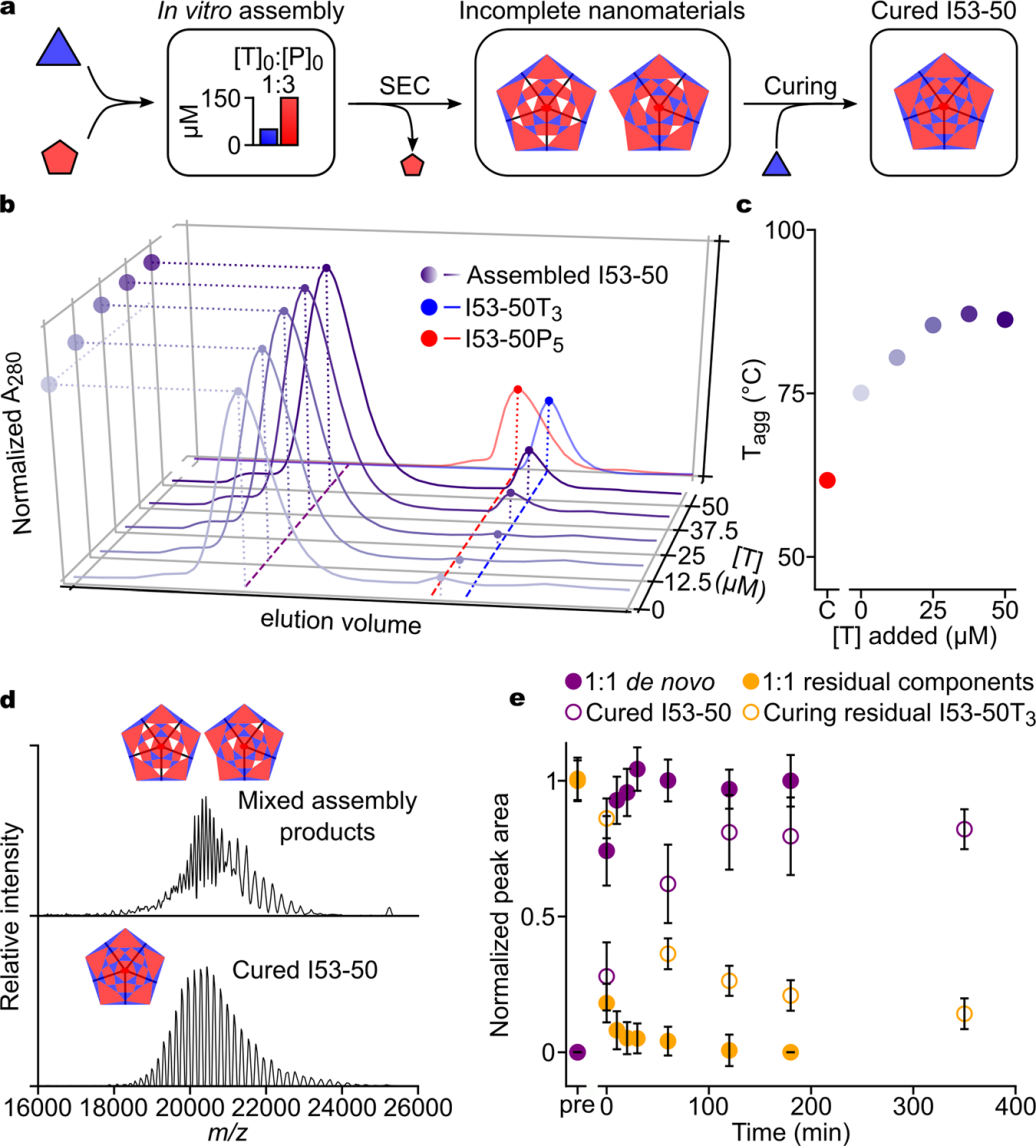
Curing of incomplete I53-50 assemblies by post-assembly addition of trimer. **a** Schematic for aberrant assembly subsequently cured by addition of free trimer. **b** SEC analysis of curing reactions, where shades of purple indicates the amount of free trimer added. **c** *T*_agg_ of SEC-purified cured nanomaterials. The concentration of the uncured aberrant assembly (0 μM added T) was adjusted such that its absorption at 280 nm signal was equivalent to 70 μM nanomaterial assembled at [T]_0_ = [P]_0_. **d** Native MS of incomplete assembly reaction after SEC (top) displaying at least two charge state series. After trimer addition, the spectrum shows a distinct, single charge state series corresponding to the complete 120-subunit nanomaterial (bottom). **e** SEC analysis of de novo assembly reactions at [T]_0_ = [P]_0_ = 12.5 μM (filled circles) and curing reactions comprising 12.5 μM additional T (open circles) incubated for varying amounts of time. Average peak area is normalized to reaction endpoints. Error bars are the standard deviation of three replicates. Source data are provided as a Source Data file.

Although the two-component systems studied here generally behaved highly cooperatively and assembled into well-defined complexes, the I53-50 assemblies showed deviating behavior in some experimental regimes. Our cooperativity and biophysical analyses (Figs. 1 and  2) suggested that the aberrant I53-50 assemblies obtained from reactions where [P]_0_ > [T]_0_ were either incomplete on-pathway assemblies lacking the full complement of trimeric building blocks, or complete assemblies that packaged or otherwise irreversibly bound excess pentameric component. To distinguish between these two possibilities, we tested whether the addition of trimeric component post-assembly could “cure” these potentially incomplete assemblies to yield 120-subunit structures. As increasing amounts of added T (0, 12.5, 25, and 50 μM) were provided to SEC-purified aberrant assemblies produced by in vitro assembly reactions with [T]_0_ = 50 μM and [P]_0_ = 150 μM (Fig. 5a), we observed slight increases in the peak area of the assembled nanomaterial peak, suggesting incorporation of the trimeric building blocks into the assemblies (Fig. 5b). No residual trimer was observed until >25 μM T was added. Determination of *T*_agg_ for the cured nanomaterials revealed that trimer addition had a stabilizing effect that plateaued between 12.5 and 37.5 μM additional T, with stability of fully cured structures similar to that obtained earlier for complete, 120-subunit assemblies (Fig. 5c). We observed similar results when we provided additional trimer directly to aliquots of the same in vitro assembly reactions without first purifying the assemblies by SEC (Supplementary Fig. 6c–e). Native MS confirmed that the cured assemblies were indeed complete, 120-subunit complexes (Fig. 5d). Together, these data demonstrate that the aberrant assemblies formed when [P]_0_ > [T]_0_ are incomplete, on-pathway assemblies lacking the full complement of trimeric building blocks, and that these assemblies can be completed through post-assembly addition of trimeric components. We suggest that—if the distribution of trimeric voids is not random, which may be expected based on considerations of the stability of such assembly intermediates—this feature may provide a route to asymmetrically functionalizing the materials through controlled multi-stage decoration, analogous to the filling of a molecular breadboard^53^.

Stable, on-pathway partial assemblies may arise because of kinetic rather than thermodynamic effects^50,54^. Equimolar assemblies of I53-40 and I53-50 proceeded at similar overall rates as determined by SLS (Supplementary Fig. 6a, b), indicating that the propensity of I53-50 to become kinetically trapped cannot be ascribed merely to faster assembly kinetics. Rather, the observed asymmetry in assembly products with respect to the two components of I53-50—that partial assemblies appear to specifically lack trimeric components and only occur when the trimer component is limiting ([P]_0_ > [T]_0_)—suggested that the addition of trimeric building blocks to nearly complete assemblies may be significantly slower than nucleation of new assemblies and the early stages of their growth. To test this hypothesis, we compared the increase in the assembled nanomaterial peak by SEC over time for two types of reactions: (i) de novo I53-50 in vitro assembly reactions with [T]_0_ = [P]_0_ = 12.5 μM and (ii) curing reactions in which SEC-purified partial assemblies were provided with 12.5 μM additional T. The increase in the assembled nanomaterial peak (and corresponding depletion of the residual component peak) was rapid in the de novo reactions, exceeding half its maximum value within the experimental dead time (about 15 min), whereas the curing reactions required an additional 60 min beyond the dead time, providing direct evidence for comparatively slow addition of trimeric building blocks to nearly complete assemblies (Fig. 5e).

## Discussion

Previous observations of complex, non-cooperative folding of de novo designed proteins^39^, along with parallels drawn between cooperative virus capsid assembly and two-state intramolecular folding^21^, led us to investigate the assembly of computationally designed protein nanomaterials that lack an evolutionary history. We observe that cooperative assembly can be intrinsic to hierarchically structured protein nanomaterials, but the non-cooperative assembly observed for I53-50 in certain regimes indicates it is a conditional rather than a universal feature.

What causes this difference between I53-40, which assembles cooperatively in all regimes tested, and I53-50? Structural metrics from the computational design models do not implicate major differences in the two designed interfaces^6^. Nevertheless, the relatively high contact energy of I53-50 is an established correlate of kinetic trapping, a phenomenon which arrests assembly progress and “locks in” deviations from the target structure. Because equimolar assemblies proceed at similar apparent rates for both nanomaterials, it is possible that partial I53-40 structures transiently form in some non-stoichiometric regimes, but then (unlike I53-50) self-cure via disassembly and error correction prior to analysis. These results are consistent with previous analyses of in vitro virus capsid assembly, which suggest that relatively weak interfaces are required for cooperativity^23,33,50,54^. Furthermore, for HBV, a virus capsid architecture with thermodynamic properties similar to I53-40 and I53-50, contact energies and kinetic parameters which lead to cooperative assembly are constrained by the dual requirements of efficiency and fidelity^33,55^. This raises the possibility that the design of overly strong inter-building block interfaces may be a previously unappreciated failure mode in computational design of novel self-assembling proteins. If so, our data point to a new route to improve these computational methods.

We speculate that two structural features contribute to the intriguing asymmetry of the I53-50 assembly data with respect to subunit stoichiometry. The first is topological: the I53 architecture can evenly distribute up to seven trimeric voids over the nanomaterial surface while maintaining at least three inter-subunit contacts per oligomeric component. Pentameric void(s) by contrast necessitate that the five adjacent trimers are held in place by only two contacts. This difference may accelerate disassembly of structures that lack pentamers and subsequent equilibration to the target thermodynamic product. Alternatively, trimeric defects may arise from the particular morphology of the I53-50 nanomaterial: in assembled I53-50, the trimeric components are recessed relative to the pentamer, closer to the geometric center of the material (see Fig. 1b). This may sterically hinder the addition of trimeric components unless access to the nanomaterial lumen is provided via a contiguous void of multiple missing components. Indeed, kinetic data show that incomplete I53-50 assemblies can be cured by addition of trimer, but this completion pathway is slower than that observed for equimolar de novo assembly, despite comprising fewer steps. Imbalanced subunit stoichiometry (specifically, where [P]_0_ > [T]_0_) therefore must direct assembly toward a class of structures featuring multiple discrete trimeric voids, which comprise a kinetic bottleneck. Their existence at the moment of subunit exhaustion is attributable to the interplay between input stoichiometry and the steric deceleration in the late stages of the resulting assembly pathways. Their persistence can be ascribed to higher intersubunit affinity and advantageous distribution of trimeric voids. Analysis of future cases of non-cooperative assembly in designed protein nanomaterials may enable further evaluation of these mutually compatible hypotheses.

Notably, the equilibrium model adapted here to the I53 architecture identifies a non-stoichiometric regime where incomplete structures—though low in absolute terms—are orders of magnitude higher in relative abundance than predicted for one-component architectures^21^. The magnitude of this “thermodynamic” non-cooperativity is not highly sensitive to interface strength (and happens to be dwarfed in the case of I53-50 by kinetic effects), but the prediction raises questions concerning what quantitative thresholds ought to drive the practical distinction between cooperative and non-cooperative assembly. Our observations here indicate that for two-component structures assembled via a single symmetrically repeated interface, cooperative assembly is normative near equimolar conditions. However, implementation of Twarock and Keef’s adaptation to the assembly of multi-layered virus-like particles indicates that the prevalence and severity of thermodynamic non-cooperativity increases with the number of unique components and interfaces comprising a given system^51,56^. It is likely that efforts to design more complex architectures will require explicit consideration of not only the target structure, but also characteristics of intermediate structures as well as the intervening assembly process.

From a biotechnological perspective, our data indicate that in vitro assembly of two-component protein nanomaterials is generally robust to significant perturbations, such as alterations in subunit stoichiometry and total concentration. Profound hysteresis of disassembly in response to dilution, as well as substantial thermal stabilization of components upon assembly, are also advantageous features. These properties will be important for manufacturing vaccines, therapeutics, and other technologies based on computationally designed nanomaterials. There are at present three vaccine candidates based on computationally designed two-component nanoparticles being manufactured under cGMP conditions for clinical trials, including two based on I53-50 (refs. ^12–14^). In addition to identifying conditions that lead to the production of fully assembled nanomaterials, our work also demonstrates that several complementary biochemical and biophysical methods can be used to characterize the products of in vitro assembly reactions in detail, and can distinguish complete from incomplete assemblies. These assays may be useful for characterization and release during manufacturing.

We draw a parallel between the technological development of computationally designed protein nanomaterials and the evolution of virus capsids and other multi-subunit complexes^20^. Unlike cooperative intramolecular folding^39^, we have verified empirically that complete and cooperative assembly is readily achieved by synthetic self-assembling proteins that are hierarchically structured and held together by relatively weak interfaces. Although this concept was a key assumption underlying the approach we developed to computationally design protein-based nanomaterials^5^, its correctness had not been definitively demonstrated until now. The ease-of-attainment of cooperativity may lend a clue to the diverse origins of suitable components of natural multi-subunit complexes^57^, and, counterintuitively, realization of asymmetrically functionalized, incomplete, labile, and/or environmentally responsive structures may require subsequent selection, perhaps for stronger interactions which effect functionally advantageous geometric irregularities or assembly dynamics^38,53,58^. As computational protein design continues to mature^59^ and our understanding of the assembly process advances, building similar dynamic features into designed protein nanomaterials should become possible.

## Methods

### Design of I53-40 trimer variants

A consensus design approach was used to first identify surface mutations predicted to enhance the stability and/or solubility of the I53-40 components. Using the Rosetta macromolecular modeling suite, the I53-40 computational design model^6^ was redesigned by allowing optimization of the identities of relatively exposed residues (defined as having a solvent accessible surface area of greater than 20 square Ångstroms), excepting polar residues (aspartate, glutamate, histidine, lysine, asparagine, glutamine, and arginine) and residues near the designed protein–protein interface between the pentameric and trimeric components. Mutations that resulted in losses of significant atomic packing interactions or side chain-backbone hydrogen bonds were discarded. A position-specific scoring matrix (PSSM) based on homologous protein sequences was used to augment the default Rosetta score function to favor residues that appear frequently at a given position in homologous proteins^60^. Multiple design trajectories were performed with varying weights on the contribution of the PSSM, and mutations to polar residues that appeared favorable across all design trajectories were selected for inclusion in the variant proteins.

### Protein expression and purification

Cloning of individual designed components and wild-type scaffolds into the pET29b+ expression vector has been previously described^6^. Chemically competent *E. coli* strain T7 Express (New England Biolabs) were separately transformed with pET29b+ carrying 2b98, 4e38, I53-40T_3_, I53-40P_5_, I53-40-bicis, 1wa3, 2obx, I53-50T_3_, I53-50B.4PT1, and I53-50A.PT1/B.4PT1-bicis. Several colonies were used to inoculate 20–60 mL of LB containing 50 μg/mL kanamycin, which was shaken overnight at 37 °C in an orbital shaker set to 185 r.p.m. The following morning, 1 L aliquots of liquid medium (LB containing 50 μg/mL kanamycin) were inoculated with 10–20 mL of preculture. Cultures were incubated at 37 °C with shaking until OD600 reached 0.4–0.6, then overexpression of the protein of interest was induced with 0.5 mM IPTG. All cultures were incubated overnight at 20 °C except for those expressing I53-40P_5_, which was incubated at 37 °C for 4–6 h followed by refrigeration at 4 °C overnight.

Unless otherwise noted, protein preparation and analyses were carried out in either “Buffer 40” (25 mM Tris pH 8.0, 250 mM NaCl, 0.75% CHAPS, 1 mM DTT) in the case of I53-40, I53-40T_3_, I53-40P_5_, 2b98, and 4e38, or “Buffer 50” (50 mM Tris pH 8.0, 500 mM NaCl, 0.75% CHAPS, 1 mM DTT) in the case of I53-50, I53-50T_3_, I53-50P_5_, 1wa3, and 2obx.

Cells were harvested by centrifugation, then resuspended in the appropriate binding buffer supplemented with 20 mM imidazole, 1 mM phenylmethanesulfonyl fluoride, 10 μM lysozyme, and 1 μM DNAse (lysis buffer for I53-40P_5_ was supplemented with 100 mM additional NaCl). Cells were lysed by sonication and cleared lysate obtained by centrifugation and filtration of supernatant through a 5-micron syringe filter (Millipore).

Protein of interest was purified from lysates via immobilized metal-affinity chromatography (IMAC) using pre-equilibrated 5 mL HisTrap Fast Flow columns (GE Healthcare). All chromatography was performed on an AKTA Pure 25 M FPLC. After application of lysate, HisTrap columns were washed with 50–100 mL of appropriate buffer supplemented with 25 mM imidazole. To elute protein of interest, the [imidazole] of the running buffer was increased from 20 mM to 500 mM over the course of 20–30 min at a flow rate of 3 mL/min.

Fractions containing proteins of interest were pooled and concentrated using centrifugal filter devices (Millipore Amicon Ultra 15 10 kDa cutoff), then further purified via SEC on either a Superdex 200 10/300 gel filtration column (GE Healthcare) for oligomeric components and wild-type scaffolds, or a Superose 6 10/300 gel filtration column for assembled nanomaterials. Protein concentrations were quantified by UV-Vis spectroscopy (*A*_280 nm_). Fractions were pooled and stored at 4 °C for subsequent analyses, and designed components remained stable and assembly competent for more than 1 month.

### In vitro assembly

In vitro assembly was executed at 0.5–10 mL scale by calculating the volume of each component stock and buffer necessary to achieve a desired final concentration of each component monomer. Trimeric components were diluted into ice-cold buffer followed by addition of concentrated pentameric component and immediate mixing. These assembly mixtures were quickly removed to the bench top and allowed to stand at ambient temp (296 K) for at least 8 h where limiting component concentrations were >10 μM, or 48 h for samples containing <10 μM limiting component. Thereafter, samples were stored at 4 °C for up to 2 weeks in order to complete subsequent analyses.

### Negative stain electron microscopy

Three microliters of SEC-purified I53-40 or I53-50 (1:1 [T]_0_:[P]_0_), diluted to 100 µg/mL in 50 mM Tris pH 8.0, 500 mM NaCl, 0.75% CHAPS, 1 mM DTT or 25 mM Tris pH 8.0, 250 mM NaCl, 0.75% CHAPS, 1 mM DTT, respectively, was applied to a freshly glow-discharged 300 mesh Cu grid (Ted Pella, Catalog # 1GC300) and incubated for 1 min. The grid was submerged in a 50 µL droplet of water, then excess liquid was blotted away with filter paper (Cytiva, Catalog #1004047). Next, the grid was dipped into a 6 µL droplet of 0.75% w/v uranyl formate stain and again blotted. Finally, the grid was incubated with another 6 µL of stain for ~80 s. After a final blot to remove excess stain, the grid was allowed to dry for 1 min prior to imaging or storing. Each grid was imaged at 57,000× magnification using a Talos L120C electron microscope equipped with a Gatan camera.

### SEC analysis

Samples were centrifuged at 13,000 × *g* for 5 min prior to analysis by SEC. Volumes ranging from 0.1 to 2 mL were injected onto a Superose 6 10/300 GL column. Chromatograms were obtained at room temperature with a 0.5 mL/min flow rate while monitoring absorbance at 280 nm; 1 mL fractions were collected throughout the study. Each nanomaterial eluted in a symmetrical peak approximately 3–4 mL wide, and the 1 mL fraction straddling the peak maximum was selected for subsequent biophysical characterization.

### PAGE analysis

Assembled nanomaterials, designed components, and wild-type scaffolds were normalized to 20 μM total monomer prior to addition of SDS-PAGE loading dye containing beta-mercaptoethanol. Residual free component fractions were pooled without concentration adjustment. Concentration-normalized samples were heated to 95 °C for 10 min in a thermal cycler (BioRad) prior to loading 50 pmol (monomer equivalent) of total protein onto a 4–20% Criterion TGX precast gel (BioRad). I53-40 residual unassembled component fractions were diluted and treated similarly, while about 2× more sample volume was loaded in each lane for corresponding I53-50 samples due to their lower [protein]. Gel running buffers were prepared by diluting 10× Tris/Glycine/SDS (BioRad). All samples were subjected to electrophoresis at 50 V for 30 min, followed by 150 V for about 1 h. Gels were stained with GelCode Blue (Pierce) for not less than 2 h followed by destaining in DI water overnight. Native PAGE analysis was conducted similarly, except that denaturation by SDS, reduction, and heat were omitted from the protocol. Samples in the native state were subjected to electrophoresis in appropriately diluted 10× Tris/Glycine (BioRad) running buffer at 50 V for 30 min, followed by 150 V for about 3 h.

### Treatment of experimental in vitro assembly data

Peaks in SEC chromatograms resulting from 1 mL injections were automatically integrated (Unicorn Evaluation Classic), followed by minor manual adjustments to eliminate split peaks and misplaced baseline nodes. Summed peak area for nanomaterial and residual unassembled component(s) obtained from equimolar 50 μM assemblies were slightly higher than the summed peak area when 50 μM of each component was injected separately. We hypothesize that increased scattering in the assembled state accounted for the excess *A*_280 nm_ signal (~10% additional signal for both I53-40 and I53-50 nanomaterials compared with components injected separately). To account for this difference in signal between assembled and non-assembled material, nanomaterial peak areas were normalized to the average summed signal (for all peaks in SEC trace) obtained upon injection of 50 μM equimolar assemblies, whereas unassembled component peak areas were normalized to the sum of two separate 50 μM component injections.

### Native MS data acquisition

For native MS, I53-40 and I53-50 were assembled by mixing equal volumes of trimer and pentamer at 1:1, 1:3, and 3:1 molar [T]_0_:[P]_0_ ratios. Assembly reactions were carried out at room temperature for 4 h. All samples were buffer exchanged into aqueous ammonium acetate solution (200 mM and 500 mM at pH 8 for I53-40 and I53-50, respectively) using Micro Bio-Spin P6 columns (Biorad) following the manufacturer’s procedure for buffer exchange. An aliquot of 1–2 μL was loaded into gold-coated borosilicate capillaries 467 (prepared in-house) for nano-ESI. Samples were analyzed on a standard commercial Q Exactive-UHMR instrument (Thermo Fisher Scientific)^61,62^. Instrument parameters were optimized for the transmission of high mass ions. Therefore, ion transfer target *m/z* and detector optimization was set to “high *m/z*”, in-source trapping was enabled with a desolvation voltage of −50 V, and the ion transfer optics (injection flatapole, inter-flatapole lens, bent flatapole, and transfer multipole) were set to 10, 10, 4, and 4 V, respectively. Xenon was used as collision gas for all experiments at about 2 × 10^−9^ mbar (UHV readout) and particles were desolvated in the HCD cell with HCD energies ranging between 100 and 130 V. For tandem MS experiments, charge state distributions were isolated in the quadrupole and subunit were ejected with HCD energies ranging between 180 and 200 V. Data were recorded at resolution settings corresponding to 32 ms transients and scans were summed in the Xcalibur Qualbrowser to the final displayed mass spectra.

### Circular dichroism (CD)

Samples were diluted or concentrated to 20 μM total monomer in the appropriate buffer containing fresh DTT. CD analysis was carried out on a JASCO J-1500, using a 1 mm quartz cuvette. Samples were heated at a ramp rate of 1 °C/min while monitoring ellipticity at 222 nm. Data were collected at 1 °C intervals without pausing temperature ramping. Data integration time was 2 s, bandwidth was 1 nm.

### Integrated nanoDSF, DLS, and SLS

Sample concentrations were normalized to 10 μM total monomer. DLS data were collected using an UNcle instrument (UNchained Labs), with ten 5-s acquisitions. Average hydrodynamic diameter for each sample was calculated using the UNcle software. Data for two technical replicates were checked for correspondence and then averaged. SLS and intrinsic tryptophan fluorescence (presented as the barycentric mean (BCM) of the emission spectrum) were measured on the same samples in triplicate at 25 °C followed by a thermal ramp from 50 °C to 95 °C at a ramp rate of 1.0 °C/min.

### *T*_m_ and *T*_agg_ determination

Melting point (*T*_m_) determination was performed by applying a quadratic interpolation to the I53-40 CD and enzyme activity data, and then locating the maximum of the derivative of the resulting function using the python tools scipy.interpolate.interp1d and numpy.diff. For all other datasets (nanoDSF, SLS, and I53-50 CD), *T*_m_ or *T*_agg_ was determined by setting an arbitrary signal threshold and then calculating the temperature at which a cubic interpolation of the dataset exceeded that threshold. Equations describing threshold lines were constructed by setting slope equal to the average baseline drift in the whole dataset, and setting the *y*-intercept (value of threshold line at the starting temperature) based on a fraction of a typical endpoint signal. For example, for the I53-50 CD data, this intercept was 20% of the expected loss of ellipticity due to denaturation of unassembled pentamer. For SLS data, about 60,000 cts over background was a typical endpoint obtained for analysis of I53-40 and I53-50, therefore the intercept was set to 20% of this value. For nanoDSF the intercept was set to 50% and 20% of the shift observed at 95 °C for I53-40 (−4.5 nm) or I53-50P_5_ (−14.2 nm), respectively.

### Aldolase activity assay

SEC-purified nanomaterials, as well as the trimeric aldolases I53-40T_3_ and 4e38 were diluted into “40 buffer” lacking CHAPS such that the total aldolase concentration was 10 μM; 100 μL aliquots of these samples were subjected to a range of incubation temperatures for 1 h. After incubation, samples were centrifuged at 5000 × *g* for 1 h and then diluted to 0.1 μM. Aldolase activity was measured via an LDH-coupled colorimetric assay. A master mix containing 0.1 mg/mL L-LDH (Sigma), 0.1 mM NADH (Sigma), 1 mM 2-keto-3-deoxy6-phosphogluconic acid (KDPG, Sigma) was prepared and 90 μL aliquots were added to a Corning UV transparent half-area 96-well plate. Ten microliters of diluted aldolase sample was added to each well to initiate the coupled reaction, and the consumption of NADH was monitored via *A*_339 nm_ on a SpectraMax M5e (Molecular Devices). The relative initial reaction velocity over 100–200 s was calculated via regression analysis (Softmax Pro).

### Hysteresis of disassembly

Several assembly reactions were prepared at 2–20 mL scale. One I53-40 assembly comprised 100 μM of each monomer (I53-40P and I53-40T). The second I53-40 assembly comprised 5 μM of each subunit. Aliquots of each assembly were removed for quantitative analysis via SEC at various time points. An aliquot of the 100 μM assembly was diluted 20-fold in Buffer 40 after 42 h of assembly. For SEC analysis, the 100 μM samples were injected onto a Superose 6 sizing column using a 0.1 mL sample loop while the 5 μM samples were injected using a 2 mL sample loop, thus normalizing the total amount of protein injected and enabling direct comparison of peak area.

### Modeling

Keef and Twarock’s adaptation^51^ of Zlotnick’s equilibrium model^21^ was used (Jupyter Notebook 6.0.3 and Python 3.7.6) to predict arrays of concentrations of partially and/or fully assembled nanoparticle dependent upon input arrays of residual unassembled [T] and [P] (in μM). In their adaptation, the term 1/s accounts for the symmetry of the on-pathway intermediates, and in the equation below for the molar concentration of full nanomaterial structure T_60_P_60_, 1/s is taken to equal 1/60 after the icosahedral examples of virus capsids detailed by Twarock and Keef. The output of this equation ([T_60_P_60_], Eq. 4) was then multiplied by 120,000,000 to yield the concentration of assembled component monomer(s) as a function of free component concentrations expressed in μM, Δ*G*_con_, and temperature (in all experiments and models temperature was 296 K).

4 $$[{\mathrm{T}}_{60}{\mathrm{P}}_{60}] = \left( {\frac{{3^{20} \times 5^{12} \times \left( {\frac{{[{\mathrm{T}}]}}{{3,000,000}}} \right)^{20} \times \left( {\frac{{[{\mathrm{P}}]}}{{5,000,000}}} \right)^{12} \times e^{\left( {60\, \times\, {{\Delta }}G_{{\mathrm{con}}}/ - R \times 296\,{\mathrm{K}}} \right)}}}{{60}}} \right)$$

For modeling I53-40 and I53-50 free component concentrations, linearly spaced arrays of values were input into a system of equations similar to the above (Supplementary Table 3). For the simplified model (equimolar assembly datasets modeled in two dimensions), only target architecture (T_60_P_60_) and free component oligomers were included in the system of equations. The inclusive model allowed for assemblies with divergent component stoichiometries, including equations for early (T_3_P_5_, T_6_P_5_, T_3_P_10_, T_3_P_15_, and T_6_P_10_) and late (T_60_P_55_, T_57_P_60_, and T_54_P_60_) on-pathway intermediates. These particular structures were chosen based on Zlotnick’s observation that the most abundant intermediates at equilibrium should reside near the termini of assembly pathways^21^. See equations below for the high MW species T_57_P_60_ (Eq. 5) and the low MW species T_3_P_5_ (Eq. 6).

5 $$[{\mathrm{T}}_{57}{\mathrm{P}}_{60}] = \left( {\frac{{3^{19} \times 5^{12} \times \left( {\frac{{[{\mathrm{T}}]}}{{3,000,000}}} \right)^{19} \times \left( {\frac{{[{\mathrm{P}}]}}{{5,000,000}}} \right)^{12} \times e^{\left( {57\, \times\, {{\Delta }}G_{{\mathrm{con}}}/ - R \times 296\,{\mathrm{K}}} \right)}}}{3}} \right)$$

6 $$[{\mathrm{T}}_3{\mathrm{P}}_5] = \left( {\frac{{3^1 \times 5^1 \times \left( {\frac{{[{\mathrm{T}}]}}{{3,000,000}}} \right)^1 \times \left( {\frac{{[{\mathrm{P}}]}}{{5,000,000}}} \right)^1 \times e^{\left( {1\, \times\, {{\Delta }}G_{{\mathrm{con}}}/ - R \times 296\,{\mathrm{K}}} \right)}}}{1}} \right)$$

The earlier, low MW on-pathway intermediates were summed (on a monomeric basis) with residual unassembled components because these species would be expected to elute rather late during SEC. Likewise, the larger on-pathway intermediates are summed into a high MW fraction along with the complete 120-subunit architecture. Residual unassembled monomer concentrations are summed with the output of the model equations to give total monomer [T]_0_ and [P]_0_ (Eq. 7, Eq. 8).

7 $$\begin{array}{l}[{\mathrm{T}}]_0 = 60[{\mathrm{T}}_{60}{\mathrm{P}}_{60}] + 60[{\mathrm{T}}_{60}{\mathrm{P}}_{55}] + 57[{\mathrm{T}}_{57}{\mathrm{P}}_{60}] + 54[{\mathrm{T}}_{54}{\mathrm{P}}_{60}] + \\ 6[{\mathrm{T}}_6{\mathrm{P}}_{10}] + 6[{\mathrm{T}}_6{\mathrm{P}}_5] + 3[{\mathrm{T}}_3{\mathrm{P}}_{15}] + 3[{\mathrm{T}}_3{\mathrm{P}}_{10}] + 3[{\mathrm{T}}_3{\mathrm{P}}_5] + [{\mathrm{T}}]\end{array}$$

8 $$\begin{array}{l}[{\mathrm{P}}]_0 = 60[{\mathrm{T}}_{60}{\mathrm{P}}_{60}] + 55[{\mathrm{T}}_{60}{\mathrm{P}}_{55}] + 60[{\mathrm{T}}_{57}{\mathrm{P}}_{60}] + 60[{\mathrm{T}}_{54}{\mathrm{P}}_{60}] + \\ 10[{\mathrm{T}}_6{\mathrm{P}}_{10}] + 5[{\mathrm{T}}_6{\mathrm{P}}_5] + 15[{\mathrm{T}}_3{\mathrm{P}}_{15}] + 10[{\mathrm{T}}_3{\mathrm{P}}_{10}] + 5[{\mathrm{T}}_3{\mathrm{P}}_5] + [{\mathrm{P}}]\end{array}$$

These three-dimensional data are forced into two dimensions by excluding all data points outside a 1 μM slice centered about the plane of interest (the [T]_0_ = [P]_0_ plane for equimolar assemblies, and the [constant component]=50 μM plane for the cooperativity analyses), then averaging the values of points aggregated within bins of 0.5 μM width (Fig. 4 and Supplementary Fig. 5). In only one circumstance in the concentration regimes modeled here was an on-pathway intermediate predicted to accumulate above 10% mass fraction. This was true where [P]_0_ > [T]_0_ throughout a range of Δ*G*_con_ values and total component concentrations for the specific intermediate T_57_P_60_. Curiously, the best-fit Δ*G*_con_ values for both I53-40 and I53-50 generated equilibrium models which predicted approximately the same amounts of this particular intermediate species. So while the model is correct in predicting the most likely class of non-target structure (on-pathway intermediates specifically lacking one or more trimers) and the conditions under which it manifests, it fails to accurately predict the mass fraction of aberrant structure(s) observed or to correlate their likelihood to differences in Δ*G*_con_. Taking these observations into account, the output of the model generally resembles a cooperative process with minor deviations under limited circumstances.

Normalization of the inclusive model outputs for comparison with normalized peak areas from SEC analysis was performed as follows. (i) Summed high MW material concentrations were normalized to the expected signal (Eq. 9) for complete nanomaterial containing 50 μM of each component. Model predicted signal was calculated by multiplying the total concentration of each component (on a per monomer basis) with the extinction coefficients of each component (Supplementary Table 4). (ii) Low MW signal was additionally multiplied by an adjustment factor accounting for the observed systematic difference (described above) between assembled nanomaterial and unassembled components (Eq. 10). The adjustment factor *F*_A280nm_ was calculated by averaging the summed *A*_280 nm_ signal obtained for the two components injected separately, and dividing by the average summed signal obtained for all species from a sample injected post-assembly (Eq. 11).

9 $${\mathrm{signal}}_{{\mathrm{high}}\,{\mathrm{MW}},\,{\mathrm{normalized}}} = \frac{{[{\mathrm{T}}]_{{\mathrm{high}}\,{\mathrm{MW}}} \times {\it{\epsilon }}_{{\mathrm{trimer}}} + [{\mathrm{P}}]_{{\mathrm{high}}\,{\mathrm{MW}}} \times {\it{\epsilon }}_{{\mathrm{pentamer}}}}}{{50\, \upmu {\mathrm{M}} \times {\it{\epsilon }}_{{\mathrm{trimer}}} + 50\, \upmu {\mathrm{M}} \times {\it{\epsilon }}_{{\mathrm{pentamer}}}}}$$

10 $${\mathrm{signal}}_{{\mathrm{low}}\,{\mathrm{MW}},\,{\mathrm{normalized}}} = F_{A280\, {\mathrm{nm}}}\left( {\frac{{[{\mathrm{T}}]_{{\mathrm{low}}\,{\mathrm{MW}}} \times {\it{\epsilon }}_{{\mathrm{trimer}}} + [{\mathrm{P}}]_{{\mathrm{low}}\,{\mathrm{MW}}} \times {\it{\epsilon }}_{{\mathrm{pentamer}}}}}{{50\, \upmu {\mathrm{M}} \times {\it{\epsilon }}_{{\mathrm{trimer}}} + 50\, \upmu {\mathrm{M}} \times {\it{\epsilon }}_{{\mathrm{pentamer}}}}}} \right)$$

11 $$F_{A280\, {\mathrm{nm}}} = \frac{{{\mathrm{sig}}_{{\mathrm{trimer}},\,50\, \upmu {\mathrm{M}}} + {\mathrm{sig}}_{{\mathrm{pentamer}},\,50\, \upmu {\mathrm{M}}}}}{{{\mathrm{sig}}_{{\mathrm{high}}\,{\mathrm{MW}},\,50\, \upmu {\mathrm{M}}\,{\mathrm{assembly}}} + {\mathrm{sig}}_{{\mathrm{low}}\,{\mathrm{MW}},\,50\, \upmu {\mathrm{M}}\,{\mathrm{assembly}}}}}$$

For I53-40, this adjustment factor was 0.89 ± 0.06 and for I53-50 it was 0.90 ± 0.04. For the series of equimolar assemblies, the mass fraction assembled nanomaterial was calculated from peak area ratios (taking into account the above adjustment factors) and converted into molar units based on the initial total component concentration, assuming that stoichiometries were equimolar in both the assembly product and residual unassembled free component fractions.

Error bars always represent the standard deviation of three technical replicates. Since all signals are normalized to the average of six signals obtained from SEC analysis of equimolar (50 μM each component) assemblies, the standard deviation of that dataset must be taken into account (Eq. 12, Eq. 13).

12 $${\mathrm{StdDev}} = \left| f \right|\sqrt {(\sigma X/X)^2 + (\sigma E/E)^2}$$

13 $$f(X) = X/E$$

where *σX* and *σE* are the standard deviations associated with the component ratio of interest and the equimolar dataset, respectively. *X* and *E* are average signals associated with the ratio of interest and the equimolar dataset, respectively.

In the simplified model, equimolar assemblies can be plotted in two dimensions by setting [T] equal to [P]. For the purposes of RMSPE analysis, the simplified model was binned in the [T]_0_=[P]_0_ plane with a bin width of 0.5 μM for I53-40 and 0.0625 μM for I53-50. The three-dimensional outputs of the inclusive equilibrium models were represented in two dimensions as described above. For error minimization between model output and experimental data, *percent error* (Eq. 14) was calculated for each data point (cooperativity analyses) or paired data points (equimolar analyses) via the equation below. The root mean squared percent error (RMSPE) (Eq. 15) for the dataset with *n* data points was then minimized by varying only Δ*G*_con_.

14 $${\mathrm{Percent}}\,{\mathrm{error}} = 100 \times \frac{{{\mathrm{Theoretical}}\,{\mathrm{value}} - {\mathrm{Experimental}}\,{\mathrm{value}}}}{{{\mathrm{Experimental}}\,{\mathrm{value}}}}$$

15 $${\mathrm{RMSPE}} = \sqrt {\frac{{\mathop {\sum }\nolimits_1^n ({\mathrm{Percent}}\,{\mathrm{error}}_n^2)}}{n}}$$

### SLS kinetics

Samples were diluted to 20 μM and kept on ice prior to loading. A DynaStar cuvette was equilibrated to 25 °C and loaded with 5 μL of trimeric component, followed immediately by addition of pentameric component and immediate pipette mixing. The cuvette was placed in a Wyatt DynaStar instrument and SLS readings were initiated immediately, resulting in a dead time of about 10–15 s. One SLS measurement was collected every second, and blanked against summed separate readings of 10 μM component(s).

### Curing experiments

Several I53-50 assembly reactions were prepared at 1–10 mL scale, where [T]_0_ = 50 μM and [P]_0_ = 150 μM; 500–1000 μL assemblies were purified by SEC, pooling all fractions under the main high-MW peak (collected between 11–13 mL). This material was of indeterminate concentration, as the stoichiometry of the aberrant assembly product is unknown. Reckoning an average of seven missing trimers per molecule, this material was diluted to ~25 μM equivalent of “unbound” P and spiked with additional 12.5, 25, 37.5, and 50 μM T from a concentrated stock; 250 μL of this reaction mixture was subsequently purified by SEC after a 4-h incubation at 296 K. Absorbance signals were multiplied by a small dilution factor correcting for volume of added trimer stock, resulting in the chromatograms in Fig. 5.

Additionally, aliquots of the aberrant I53-50 assembly described above which had not been purified by SEC were spiked with additional 50, 100, 150, or 200 μM T from a concentrated stock; 250 μL of this reaction mixture was subsequently purified by SEC after a 4 h incubation at 296 K. Absorbance signals were multiplied by a small dilution factor correcting for volume of added trimer stock, resulting in the chromatograms in Supplementary Fig. 6.

### Capsid curing without SEC for native MS

Assembly defects in I53-50 were cured by addition of 25 μM trimeric component to SEC-purified sample which contained ~25 μM trimer and 35–40 μM pentamer in the assembled state. Before all injection steps, remaining trimeric components were removed through three consecutive dilution/concentration rounds using centrifugal filter devices with a MWCO of 300 kDa (Vivaspin, 9000 × *g*). Curing reactions (i.e., after trimer addition) were carried out for 4 h at room temperature.

### Curing kinetics

Aberrant I53-50 assembly product and I53-50P_5_ were prepared as described above. Both materials were chilled on ice and mixed with a quantity of free trimer such that unbound P and T concentrations were equal to 12.5 μM. At various time points, a 150 μL aliquot of this reaction mixture was withdrawn from ice and immediately analyzed by SEC at a system temperature of 296 K. Reference reactions containing 0 or 12.5 μM free T were performed at 296 K for 4 h to approximate reaction endpoints. Signals for each species of interest are the peak area for pertinent features of the SEC traces. Assembly product signal was blanked for all time points by subtracting initial reference signal (prior to mixing with additional T). Component signal was blanked for all time points by subtracting the final residual component reference signal. Both assembly product and free component signals were then normalized to the final reference signal and initial reference signal, respectively. This experiment was performed in triplicate. Error bars represent standard deviation.

### Reporting summary

Further information on research design is available in the Nature Research Reporting Summary linked to this article.

## Supplementary information

Supplementary Information

Reporting Summary

## Data Availability

Data supporting the findings of this manuscript are available from the corresponding authors upon reasonable request. A reporting summary for this Article is available as a Supplementary Information file. Source data are provided with this paper.

## Source data

Source Data
