# Supplementary material for: Complete and cooperative in vitro assembly of computationally designed self-assembling protein nanomaterials
Source: Nat Commun. 2021 Feb 9;12:883. doi: 10.1038/s41467-021-21251-y (PMC7873210; doi:10.1038/s41467-021-21251-y)
Supplement: Supplementary file 1 — Supplementary Information [file 41467_2021_21251_MOESM1_ESM.docx]

**Complete and cooperative *in vitro* assembly of computationally designed self-assembling protein nanomaterials**

Adam J. Wargacki^1,2^, Tobias P. Wörner^3^, Michiel van de Waterbeemd^3^, Daniel R. Ellis^1,2,4^, Albert J.R. Heck^3^ & Neil P. King^1,2^

^1^Department of Biochemistry, University of Washington, Seattle, WA, USA.

^2^Institute for Protein Design, University of Washington, Seattle, WA, USA.

^3^Biomolecular Mass Spectrometry and Proteomics, Bijvoet Center for Biomolecular Research and Utrecht Institute for Pharmaceutical Sciences, Utrecht University, Utrecht, The Netherlands.

^4^Graduate Program in Molecular and Cellular Biology, University of Washington, Seattle, WA, USA.

**SUPPLEMENTARY INFORMATION**

Supplementary Tables 1-4

Supplementary Figs. 1-6

**Supplementary Table 1 | Amino acid sequences for proteins used in this study.**

| Construct | AA sequence |
| --- | --- |
| I53-40A.1 | MRGSHHHHHHGMTKKVGIVDTTFARVDMASAAILTLKMESPNIKIIRKTVPGIKDLPVACKKLLEEEGCDIVMALGMPGKKEKDKVCAHEASLGLMLAQLMTNKHIIEVFVHEDEAKDDAELKILAARRAIEHALNVYYLLFKPEYLTRMAGKGLRQGFEDAGPARE |
| I53-40B.1 | MDDINNQLKRLKVIPVIAIDNAEDIIPLGKVLAENGLPAAEITFRSSAAVKAIMLLRSAQPEMLIGAGTILNGVQALAAKEAGADFVVSPGFNPNTVRACQIIGIDIVPGVNNPSTVEQALEMGLTTLKFFPAEASGGISMVKSLVGPYGDIRLMPTGGITPDNIDNYLAIPQVLACGGTWMVDKKLVRNGEWDEIARLTREIVEQVNPGSLEHHHHHH |
| I53-40A.1  (co-expressed) | MTKKVGIVDTTFARVDMASAAILTLKMESPNIKIIRKTVPGIKDLPVACKKLLEEEGCDIVMALGMPGKKEKDKVCAHEASLGLMLAQLMTNKHIIEVFVHEDEAKDDAELKILAARRAIEHALNVYYLLFKPEYLTRMAGKGLRQGFEDAGPARE |
| I53-40B.1 (co-expressed) | MDDINNQLKRLKVIPVIAIDNAEDIIPLGKVLAENGLPAAEITFRSSAAVKAIMLLRSAQPEMLIGAGTILNGVQALAAKEAGADFVVSPGFNPNTVRACQIIGIDIVPGVNNPSTVEQALEMGLTTLKFFPAEASGGISMVKSLVGPYGDIRLMPTGGITPDNIDNYLAIPQVLACGGTWMVDKKLVRNGEWDEIARLTREIVEQVNPGSLEHHHHHH |
| I53-50A.1PT1 | MKMEELFKKHKIVAVLRANSVEEAIEKAVAVFAGGVHLIEITFTVPDADTVIKALSVLKEKGAIIGAGTVTSVEQCRKAVESGAEFIVSPHLDEEISQFCKEKGVFYMPGVMTPTELVKAMKLGHDILKLFPGEVVGPQFVKAMKGPFPNVKFVPTGGVNLDNVCKWFKAGVLAVGVGKALVKGKPDEVREKAKKFVKKIRGCTEGSLEHHHHHH |
| I53-50B.4PT1 | MNQHSHKDHETVRIAVVRARWHAEIVDACVSAFEAAMRDIGGDRFAVDVFDVPGAYEIPLHARTLAETGRYGAVLGTAFVVNGGIYRHEFVASAVINGMMNVQLNTGVPVLSAVLTPHNYDKSKAHTLLFLALFAVKGMEAARACVEILAAREKIAAGSLEHHHHHH |
| I53-50A.1PT1  (co-expressed) | MKMEELFKKHKIVAVLRANSVEEAIEKAVAVFAGGVHLIEITFTVPDADTVIKALSVLKEKGAIIGAGTVTSVEQCRKAVESGAEFIVSPHLDEEISQFCKEKGVFYMPGVMTPTELVKAMKLGHDILKLFPGEVVGPQFVKAMKGPFPNVKFVPTGGVNLDNVCKWFKAGVLAVGVGKALVKGKPDEVREKAKKFVKKIRGCTE |
| I53-50B.4PT1  (co-expressed) | MNQHSHKDHETVRIAVVRARWHAEIVDACVSAFEAAMRDIGGDRFAVDVFDVPGAYEIPLHARTLAETGRYGAVLGTAFVVNGGIYRHEFVASAVINGMMNVQLNTGVPVLSAVLTPHNYDKSKAHTLLFLALFAVKGMEAARACVEILAAREKIAAGSLEHHHHHH |

**Supplementary Fig. 1 | SEC and DLS of I53-40 and I53-50 assembled *in vitro* at various subunit stoichiometries.** SEC chromatograms of I53-40 assemblies with **a**, [P]_0_ or **b**, [T]_0_ held constant at 50 μM. SEC chromatograms of I53-50 assemblies with **c**, [P]_0_ or **d**, [T]_0_ held constant at 50 μM. DLS data for **e**, I53-40 and **f**, I53-50 where samples assembled with [T]_0_:[P]_0_ ratios of 1:1, 2:1, and 1:2 assemblies are plotted in green and purple lines respectively, shaded according to increasing [T]_0_:[P]_0_ ratio at mixing. Dot plots represent the observed diameters of each component or assembly as a function of initial [T]_0_:[P]_0_. C, single components. Source data are provided as a Source Data file.
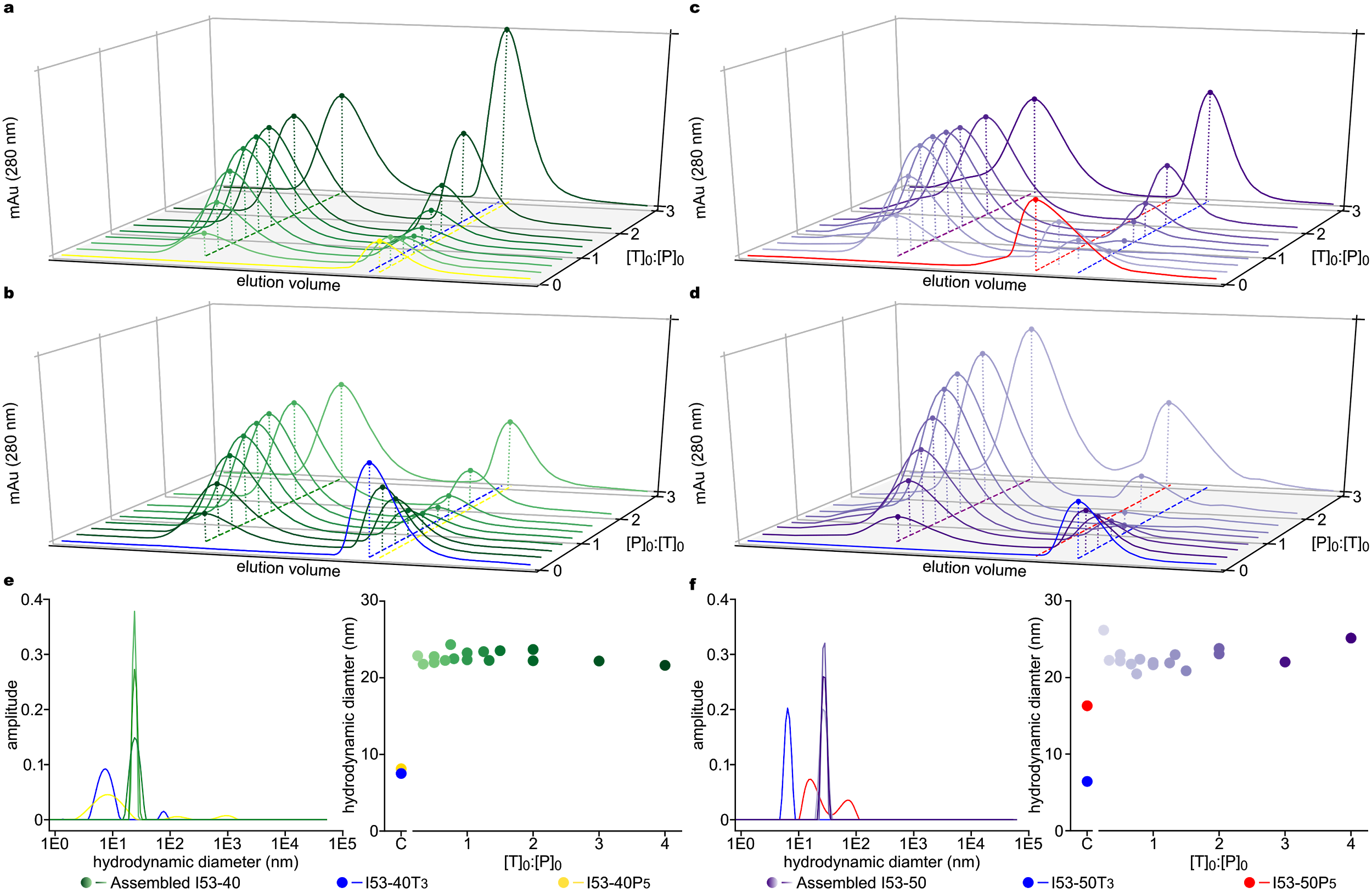


**
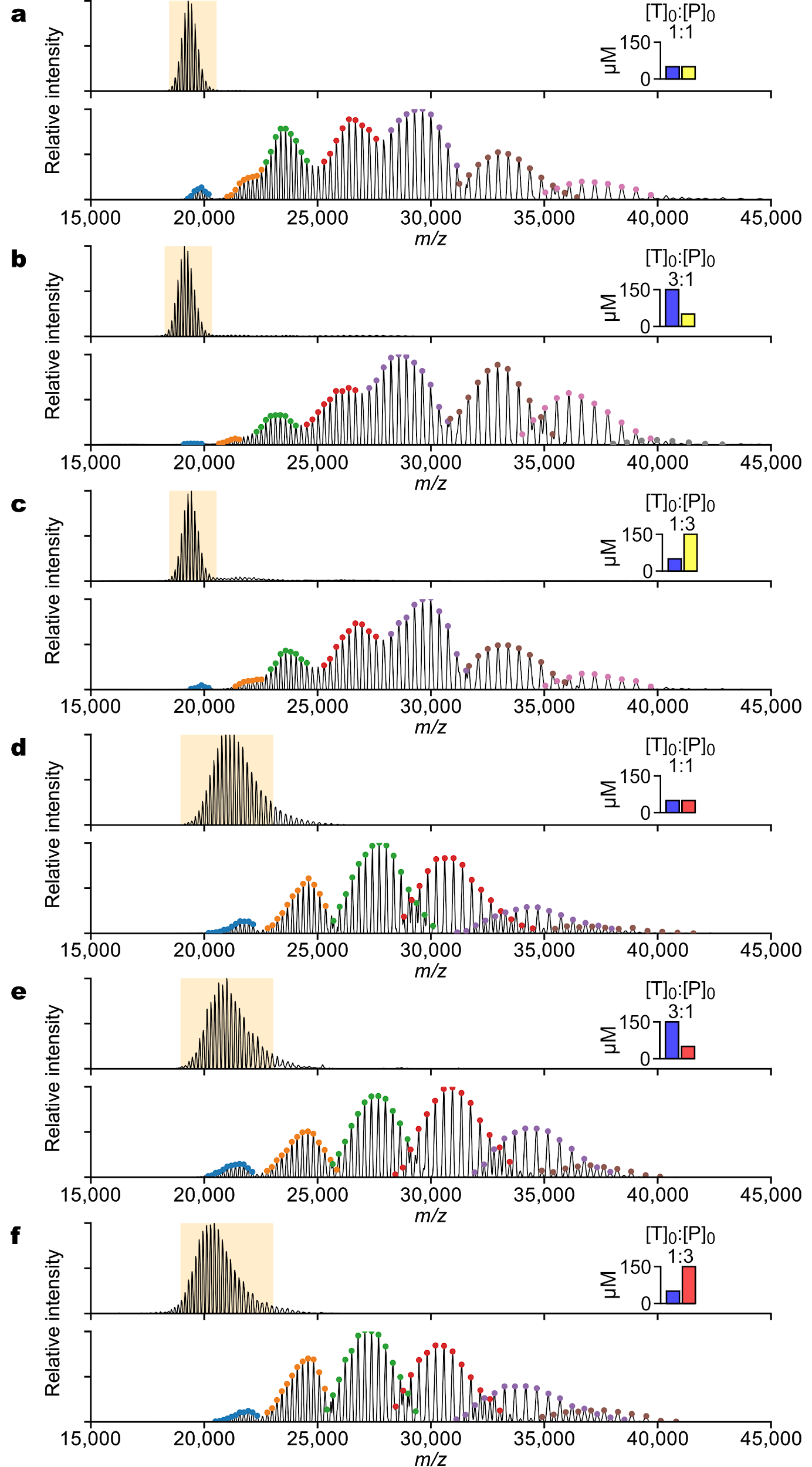
**

**Supplementary Fig. 2 | Tandem MS of assembly products unambiguously confirm their complete assembly.** Native MS spectra of complete assembly reaction products for (**a–c**) I50-40 and (**d–f**) I53-50 at varying [T]_0_:[P]_0_ ratios. Each panel shows the native mass spectra (top) alongside the corresponding tandem MS experiments (bottom). The *m/z*-range selected for fragmentation is indicated within the upper panes (orange boxes). Activation and the subsequent ejection of up to six subunits allowed in-depth characterization and confident charge state assignment. Charge states assigned to the precursor complex (blue) as well as the complex stripped of 1–6 subunits (orange, green, red, purple, brown, and pink) are indicated. Calculated and expected masses for each species are listed in **Supplementary Table 2**. Source data are provided as a Source Data file.

**Supplementary Table 2 | Expected and assigned masses for the tandem MS experiments displayed in Supplementary Fig. 2.**

|  |  | 1:1 [T]_0_:[P]_0_ | | 3:1 [T]_0_:[P]_0_ | | 1:3 [T]_0_:[P]_0_ | |
| --- | --- | --- | --- | --- | --- | --- | --- |
| Particle | Expected mass (kDa)^a^ | Measured mass (kDa) | Mass deviation^b^ | Measured mass (kDa) | Mass deviation^b^ | Measured mass (kDa) | Mass deviation^b^ |
| I53-40 | 2520 | 2525±0.3 | +0.2% | 2523±1.2 | +0.1% | 2525±0.3 | +0.2% |
| I53-40 -1sub | 2497 | 2501±0.2 | +0.2% | 2500±0.3 | +0.1% | 2501±0.5 | +0.2% |
| I53-40 -2sub | 2473 | 2477±0.2 | +0.2% | 2476±0.2 | +0.1% | 2477±0.2 | +0.1% |
| I53-40 -3sub | 2450 | 2454±0.6 | +0.2% | 2453±0.2 | +0.1% | 2453±0.5 | +0.1% |
| I53-40 -4sub | 2426 | 2430±0.2 | +0.1% | 2429±0.3 | +0.1% | 2429±0.2 | +0.1% |
| I53-40 -5sub | 2403 | 2406±0.7 | +0.1% | 2405±0.8 | +0.1% | 2406±0.7 | +0.1% |
| I53-40 -6sub | 2380 | 2382±0.5 | +0.1% | 2382±0.4 | +0.1% | 2382±0.5 | +0.1% |
| I53-50 | 2496 | 2504±0.3 | +0.3% | 2501±0.6 | +0.2% | 2502±0.5 | +0.2% |
| I53-50 -1sub | 2478 | 2485±0.2 | +0.3% | 2482±0.6 | +0.2% | 2483±0.5 | +0.2% |
| I53-50 -2sub | 2459 | 2467±0.4 | +0.3% | 2464±0.5 | +0.2% | 2464±0.5 | +0.2% |
| I53-50 -3sub | 2441 | 2449±0.2 | +0.3% | 2445±0.5 | +0.1% | 2446±0.5 | +0.2% |
| I53-50 -4sub | 2423 | 2430±0.2 | +0.3% | 2426±0.3 | +0.1% | 2427±0.4 | +0.2% |
| I53-50 -5sub | 2405 | 2412±0.3 | +0.3% | 2407±0.6 | +0.1% | 2409±0.5 | +0.2% |

^a^The average mass of the ejected subunit is 23.7 kDa and 18.6 kDa for the I53-40 and I53-50 assemblies and is in agreement with the ejection of one trimeric/pentameric subunit, respectively.

^b^Deviations from the expected mass are all well below +0.5% and can be attributed to incomplete desolvation.


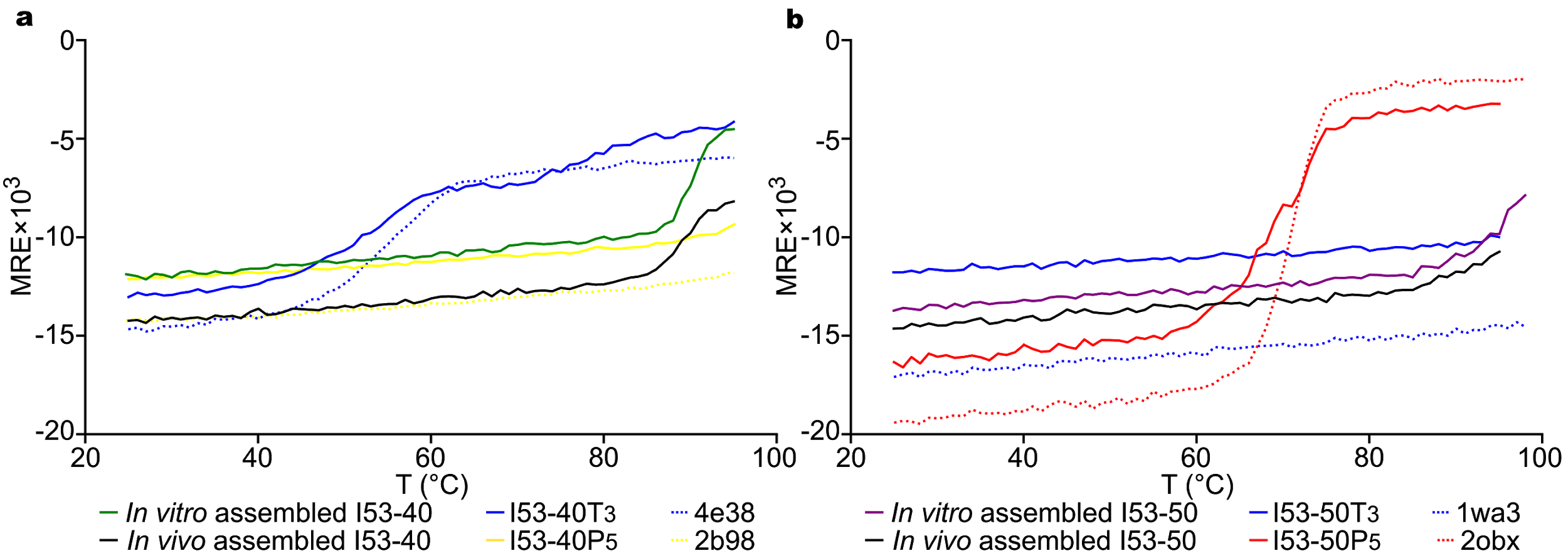


**Supplementary Fig. 3 | CD of I53-40 and I53-50 assembled *in vitro* at various subunit stoichiometries.** **a**, CD reported as MRE (mean residue ellipticity; deg×cm^2^/dmol) measured over a thermal ramp from 20–95°C for I53-40 and associated components and scaffolds. **b**, CD thermal melt for I53-50 and associated components and scaffolds. *In vivo*-assembled nanomaterials obtained via co-expression in *E. coli* in black. Source data are provided as a Source Data file.

**
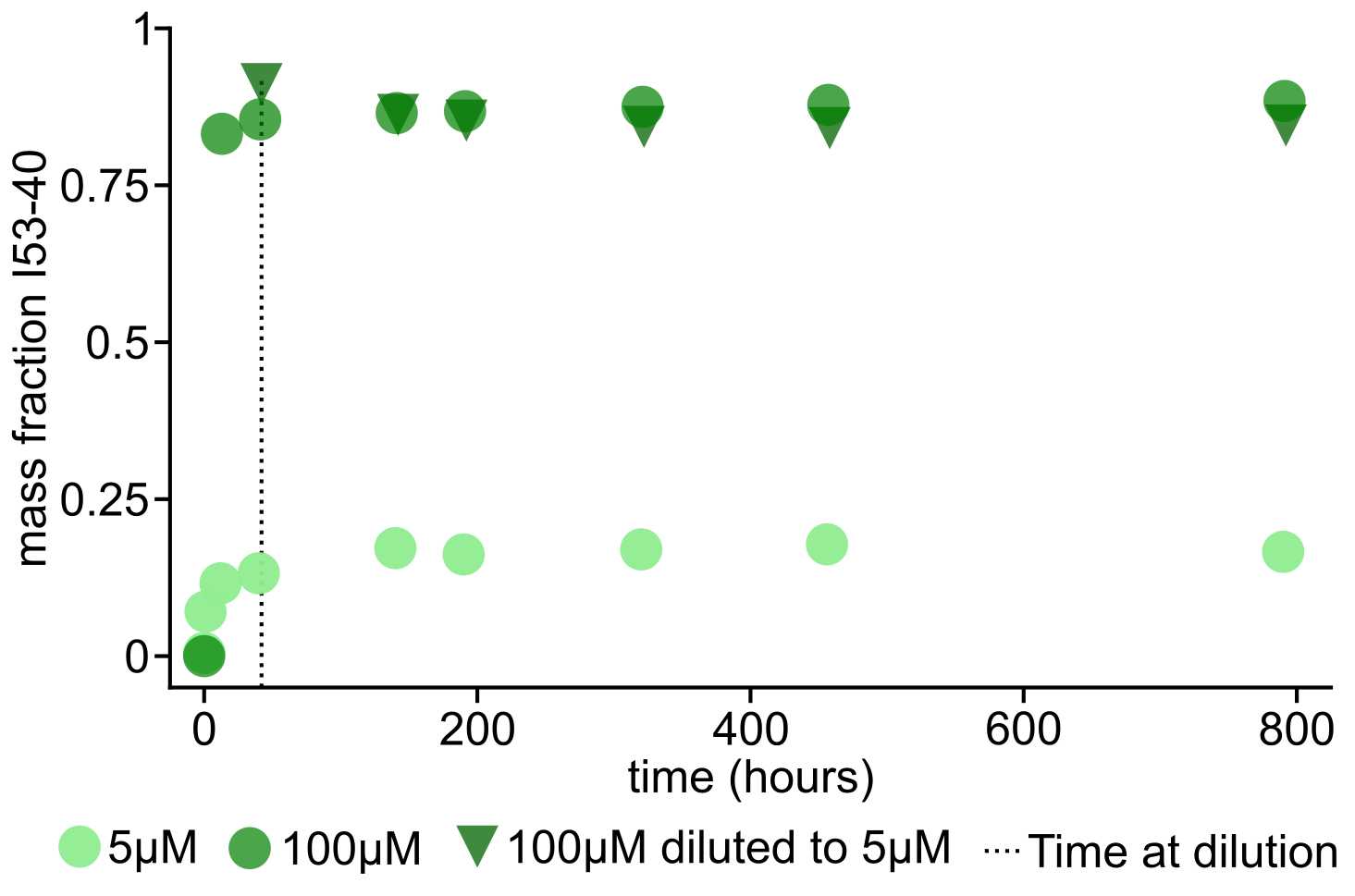
**

**Supplementary Fig. 4 | Hysteresis of I53-40 assembly.** Mass fraction nanomaterial over time was quantified by SEC for equimolar assembly reactions containing 5 μM (light green circles) or 100 μM (green circles) of each component. Dark green triangles indicate the mass fraction assembled nanomaterial for an aliquot of the 100 μM assembly diluted 20-fold after 42 h of assembly. Dotted drop line indicates time point at which 100 μM assemblies were diluted. Source data are provided as a Source Data file.

**
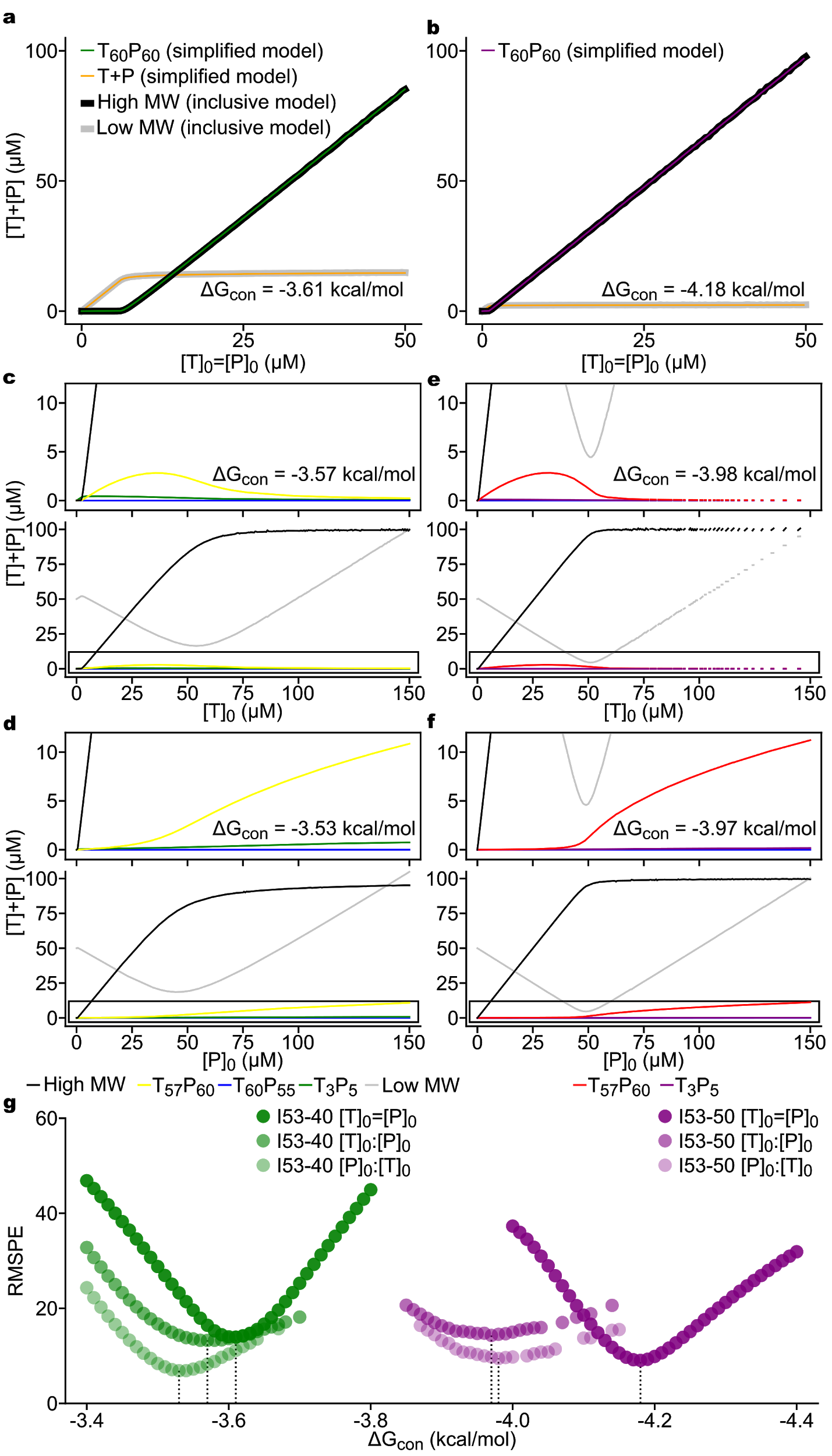
Supplementary Fig. 5 | Error minimization of model against experimental datasets. a–b**, Inclusive models binned in the [T]_0_=[P]_0_ plane are plotted as summed concentrations of structures on a per monomer basis. Thick black lines, high MW structures; thick silver lines, low molecular weight structures and unassembled components. Simplified model outputs (at the same ΔG_con_ values as the inclusive models) which take into account exclusively free components (orange) and complete 120-subunit architectures (green and purple) are overlayed as thin lines. **c–f**, Using the inclusive model, the best-fit ΔG_con_ values for the I53-40 and I53-50 cooperativity analyses are used to predict the bulk concentrations of high and low MW fractions at equilibrium. Black and silver lines represent the summed concentrations of high and low MW species (see **Methods**), respectively, on a total monomer basis. Colored lines likewise indicate the concentrations of several specific on-pathway intermediates on a total monomer basis. Upper panels of each figure magnify the plots in the low concentration range indicated by the black boxes. **g**, RMSPE minimization for various assembly analyses. At each value of ΔG_con_ (calculated in 0.01 kcal/mol increments), a Root Mean Squared Percent Error (RMSPE) is calculated between the inclusive equilibrium model and the [T]_0_:[P]_0_ and [P]_0_:[T]_0_ datasets, considering the error of each data point separately and excluding the points near the origin. For the [T]_0_=[P]_0_ datasets, error is reckoned pairwise, where for each value of [T]_0_=[P]_0_, the sum of the differences between the simplified equilibrium model and experimental values for nanomaterial and residual unassembled components are divided by the sum of the two experimental values. The ΔG_con_ where RMSPE is minimal is marked by dotted drop-lines for each dataset. Source data are provided as a Source Data file.

**
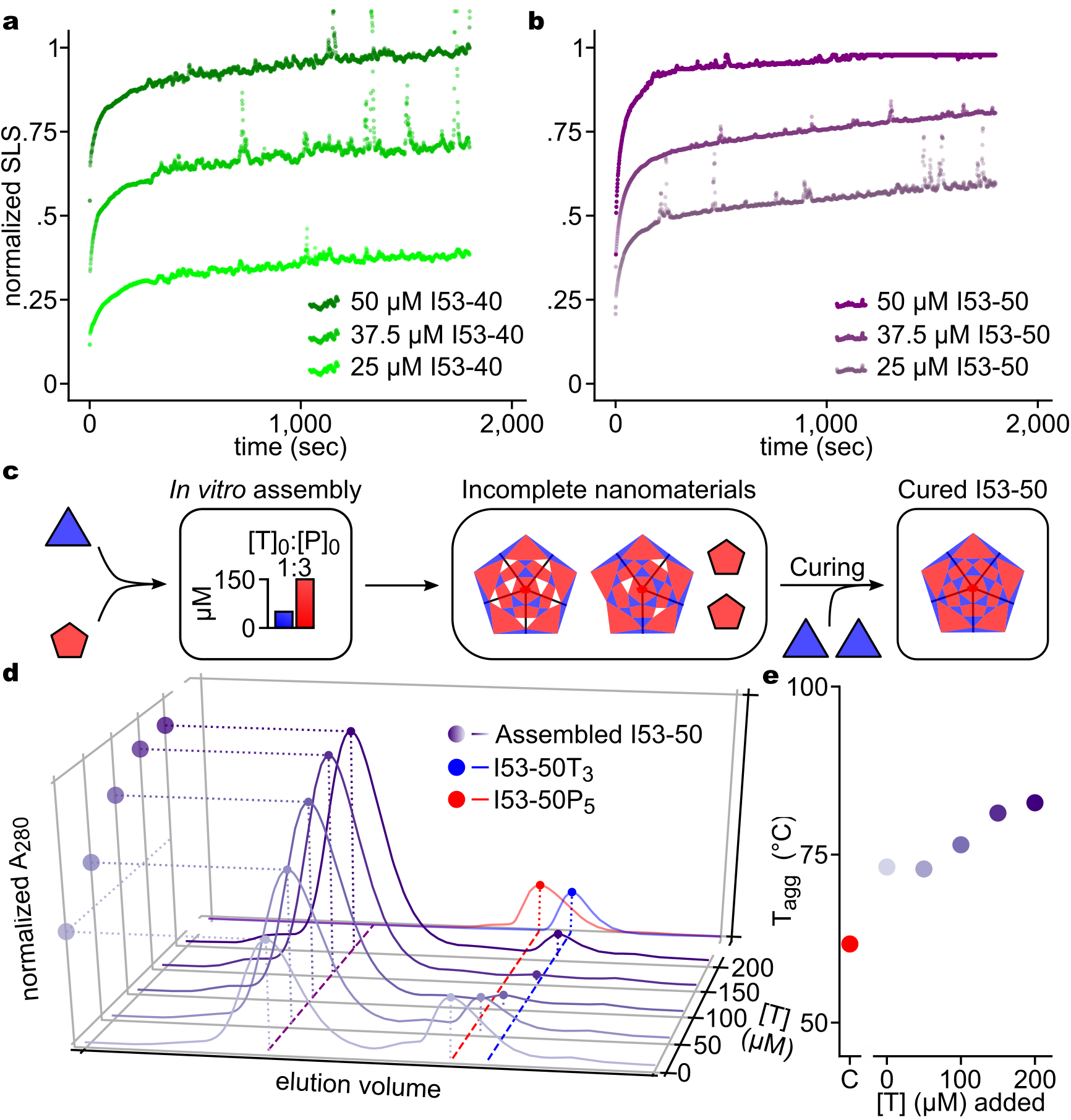
**

**Supplementary Fig. 6 | SLS kinetics for I53-40 and I53-50, Curing aberrant I53-50 nanomaterial.** **a–b**, Normalized static light scattering (SLS) as a function of time for I53-40 and I53-50 assemblies at various equimolar monomer concentrations. **c**, Schematic of curing of a non-cooperative milieu via trimer addition without an intervening SEC step, which generates complete, 120-subunit structures. Schlegel projections exemplify possible distributions of trimeric voids within the aberrant architectures. **d**, SEC traces resulting from analysis of non-cooperative milieu with addition of varying amounts of I53-50T_3_. **e**, Thermal stability, determined by the temperature at which SLS exceeds a predetermined threshold, of I53-50 nanomaterial fractions harvested from SEC experiments shown in panel **d**. Source data are provided as a Source Data file.

**Supplementary Table 3 | Examples of input arrays of component concentrations for equilibrium modeling.**

|  | I53-40  [P]_0_=50 μM | I53-40  [T]_0_=50 μM | I53-40 1:1  [T]_0_=[P]_0_ | I53-50  [P]_0_=50 μM | I53-50  [T]_0_=50 μM | I53-50 1:1  [T]_0_=[P]_0_ |
| --- | --- | --- | --- | --- | --- | --- |
| [T] (μM) | (0,105,5000)^a^ | (0,55,5000) | (0,15,10000) | (0,10,5000) | (0,55,5000) | (0,8,100000) |
| [T] (μM) | (0,10,5000) | (0,5,5000) | (4,12,10000) | (0,105,5000) | (0,0.25,5000) | (1,2,10000) |
| [P] (μM) | (0,55,5000) | (0,105,5000) | (0,15,10000) | (0,65,5000) | (0,5,5000) | (0,8,100000) |
| [P] (μM) | (0,5,5000) | (0,10,5000) | (4,12,10000) | (0 0.3,5000) | (0,105,5000) | (1,2,10000) |

^a^(x, y, z) indicates starting value, ending value, and number of indices in each array.

**Supplementary Table 4 | Extinction coefficients of I53-40 and I53-50 components.**

|  | I53-40T | I53-40P | I53-50T | I53-50P |
| --- | --- | --- | --- | --- |
| ε (M^-1^ · cm^-1^) | 14,105 | 4,595 | 6,268 | 16,025 |
