# Supplementary material for: Complete and cooperative in vitro assembly of computationally designed self-assembling protein nanomaterials
Source: Nat Commun. 2021 Feb 9;12:883. doi: 10.1038/s41467-021-21251-y (PMC7873210; doi:10.1038/s41467-021-21251-y)
Supplement: Supplementary file 2 — Reporting Summary [file 41467_2021_21251_MOESM2_ESM.pdf]

nature research

Corresponding author(s): Neil King

Last updated by author(s): Dec 20, 2020

Reporting Summary

Nature Research wishes to improve the reproducibility of the work that we publish. This form provides structure for consistency and transparency in reporting. For further information on Nature Research policies, see our Editorial Policies and the Editorial Policy Checklist.

Statistics

For all statistical analyses, confirm that the following items are present in the figure legend, table legend, main text, or Methods section.

n/a	Confirmed	
☐	☒	The exact sample size (*n*) for each experimental group/condition, given as a discrete number and unit of measurement
☐	☒	A statement on whether measurements were taken from distinct samples or whether the same sample was measured repeatedly
☒	☐	The statistical test(s) used AND whether they are one- or two-sided<br>*Only common tests should be described solely by name; describe more complex techniques in the Methods section.*
☒	☐	A description of all covariates tested
☒	☐	A description of any assumptions or corrections, such as tests of normality and adjustment for multiple comparisons
☐	☒	A full description of the statistical parameters including central tendency (e.g. means) or other basic estimates (e.g. regression coefficient) AND variation (e.g. standard deviation) or associated estimates of uncertainty (e.g. confidence intervals)
☒	☐	For null hypothesis testing, the test statistic (e.g. *F*, *t*, *r*) with confidence intervals, effect sizes, degrees of freedom and *P* value noted<br>*Give P values as exact values whenever suitable.*
☒	☐	For Bayesian analysis, information on the choice of priors and Markov chain Monte Carlo settings
☒	☐	For hierarchical and complex designs, identification of the appropriate level for tests and full reporting of outcomes
☒	☐	Estimates of effect sizes (e.g. Cohen's *d*, Pearson's *r*), indicating how they were calculated

Our web collection on statistics for biologists contains articles on many of the points above.

Software and code

Policy information about availability of computer code

Data collection	Jupyter Notebook 6.0.3. Python 3.7.6 Unicorn Evaluation Classic (Cytiva) Softmax Pro UNchained Labs Analysis Software 3.0
Data analysis	Jupyter Notebook 6.0.3. Python 3.7.6 Unicorn Evaluation Classic (Cytiva) Softmax Pro UNchained Labs Analysis Software 3.0

For manuscripts utilizing custom algorithms or software that are central to the research but not yet described in published literature, software must be made available to editors and reviewers. We strongly encourage code deposition in a community repository (e.g. GitHub). See the Nature Research guidelines for submitting code & software for further information.

Data

Policy information about availability of data

All manuscripts must include a data availability statement. This statement should provide the following information, where applicable:

- Accession codes, unique identifiers, or web links for publicly available datasets
- A list of figures that have associated raw data
- A description of any restrictions on data availability

All images and data were generated and analyzed by the authors, and are made available in the accompanying Source Data File.

Field-specific reporting

Life sciences study design

All studies must disclose on these points even when the disclosure is negative.

Sample size	Sample size for biochemical analysis was chosen to be 3 for all data that was fit to theoretical models. For biophysical analyses and native mass spectrometry, each experiment was repeated at least twice. These are consistent with current good practice in the field.
Data exclusions	Where serious instrument error occurred during analysis, data was excluded and the experiment repeated. This occurred when, on occasion, SEC runs terminated prematurely, preventing useful analysis.
Replication	Most experiments were repeated three or more times with highly similar results. No experiments could not be replicated.
Randomization	There are no experiments in this study that could have been randomized.
Blinding	There are no experiments in this study that could have been blinded.

Reporting for specific materials, systems and methods

We require information from authors about some types of materials, experimental systems and methods used in many studies. Here, indicate whether each material, system or method listed is relevant to your study. If you are not sure if a list item applies to your research, read the appropriate section before selecting a response.

Materials & experimental systems

n/a	Involved in the study	
☒	☐	Antibodies
☒	☐	Eukaryotic cell lines
☒	☐	Palaeontology and archaeology
☒	☐	Animals and other organisms
☒	☐	Human research participants
☒	☐	Clinical data
☒	☐	Dual use research of concern

Methods

n/a	Involved in the study	
☒	☐	ChIP-seq
☒	☐	Flow cytometry
☒	☐	MRI-based neuroimaging
